# Supplementary material for: Ruling out acute coronary syndrome in primary care with a clinical decision rule and a capillary, high-sensitive troponin I point of care test: study protocol of a diagnostic RCT in the Netherlands (POB HELP)
Source: BMJ Open. 2023 Jun 8;13(6):e071822. doi: 10.1136/bmjopen-2023-071822 (PMC10255045; doi:10.1136/bmjopen-2023-071822)
Supplement: Author's manuscript [file bmjopen-2023-071822.draft_revisions.pdf]

BMJ Open

BMJ Open is committed to open peer review. As part of this commitment we make the peer review history of every article we publish publicly available.

When an article is published we post the peer reviewers' comments and the authors' responses online. We also post the versions of the paper that were used during peer review. These are the versions that the peer review comments apply to.

The versions of the paper that follow are the versions that were submitted during the peer review process. They are not the versions of record or the final published versions. They should not be cited or distributed as the published version of this manuscript.

BMJ Open is an open access journal and the full, final, typeset and author-corrected version of record of the manuscript is available on our site with no access controls, subscription charges or pay-per-view fees (http://bmjopen.bmj.com).

If you have any questions on BMJ Open's open peer review process please email info.bmjopen@bmj.com

BMJ Open

Rule out of acute coronary syndrome in primary care: A clinical decision rule including a capillary, high-sensitive troponin I point of care test. Design and rationale of a clustered RCT (POB HELP)

Journal:	*BMJ Open*
Manuscript ID	bmjopen-2023-071822
Article Type:	Protocol
Date Submitted by the Author:	12-Jan-2023

Complete List of Authors:	van den Bulk, Simone; Leiden Universitair Medisch Centrum, Public Health and primary Care<br>Petrus, Annelieke; Leiden Universitair Medisch Centrum, Public Health and primary Care<br>Willemsen, Robert ; Maastricht University, Department of Family Medicine<br>Boogers, Mark; Leiden Universitair Medisch Centrum, Cardiology<br>Meeder, Joan; VieCuri Medisch Centrum voor Noord-Limburg, Cardiology<br>Rahel, Braim; VieCuri Medisch Centrum voor Noord-Limburg, Cardiology<br>van den Akker-van Marle, Elske; Leiden University Medical Center, Biomedical Data Sciences<br>Numans, Mattijs; Leiden Universitair Medisch Centrum, Public Health and primary Care<br>Dinant, Geert-Jan; Maastricht University, Department of Family Medicine<br>Bonten, Tobias N.; Leids Universitair Medisch Centrum, Public Health and primary Care

Keywords:	Myocardial infarction < CARDIOLOGY, PRIMARY CARE, Decision Making, ACCIDENT & EMERGENCY MEDICINE

SCHOLARONE™
Manuscripts

Rule out of acute coronary syndrome in primary care: A clinical decision rule including a capillary, high-sensitive troponin I point of care test

Design and rationale of a clustered RCT (POB HELP)

S. van den Bulk MD[1], A.H.J. Petrus MD PhD[1], R.T. Willemsen MD PhD[2], M.J. Boogers[3], J.G. Meeder[4], B.M. Rahel[4], M.E. van den Akker-van Marle[5], PhD, M.E. Numans MD PhD[1], G.J. Dinant MD PhD[2], T.N. Bonten MD PhD[1]

Word count: 3489

Addresses of authors

1 Department of Public Health and Primary Care
Leiden University Medical Center
Post zone V0-P
Postbus 9600
2300 RC Leiden, The Netherlands

2 Faculty of Health, Medicine and Life Sciences
Maastricht Universitair Medisch Centrum+
Capaciteitsgroep Huisartsgeneeskunde
PO BOX 616
6200 MD Maastricht, The Netherlands

3 Department of Cardiology
Leiden University Medical Center
Post zone V0-P
Postbus 9600
2300 RC Leiden, The Netherlands

4 Department of Cardiology
VieCuri Medical center for Noord-Limburg
PO BOX 1926
5900 BX Venlo, The Netherlands

5 Department of Biomedical Data Sciences,
unit Medical Decision Making
Leiden University Medical Center
Post zone J10-S
Postbus 9600
2300 RC Leiden, The Netherlands

Corresponding author

Simone van den Bulk, MD
@ s.van_den_bulk@lumc.nl
T: +31 625043440

Department of Public Health and Primary Care
Leiden University Medical Center
Post zone V0-P
Postbus 9600
2300 RC Leiden, The Netherland

For peer review only

Abstract

Introduction: Chest pain is a common reason for consultation in primary care. To rule out acute coronary syndrome (ACS), general practitioners (GP) refer 40-70% of patients with chest pain to the emergency department (ED). Only 10-20% of those referred, are diagnosed with ACS. A clinical decision rule, including a high-sensitive cardiac troponin-I point-of-care test (hs-cTnI-POCT), may safely rule out ACS in primary care. Being able to safely rule out ACS at the GP reduces unnecessary referrals and thereby alleviates the burden on the ED. Moreover, the patient receives prompt feedback which can reduce anxiety and stress.

Methods and analysis: The POB HELP study is a clustered randomised controlled diagnostic trial investigating the (cost-)effectiveness and diagnostic accuracy of a primary care decision rule for acute chest pain, consisting of the Marburg Heart Score combined with a hs-cTnI-POCT. General practices are 2:1 randomised to the intervention group (clinical decision rule) or control group (regular care). Patients with acute chest pain are planned to be included by GPs in three regions in The Netherlands. Primary endpoints are the number of hospital referrals and the diagnostic accuracy of the decision rule 24 hours, 6 weeks and 6 months after inclusion.

Ethics and dissemination: The medical ethics committee Leiden-Den Haag-Delft (the Netherlands) has approved this trial. Written informed consent will be obtained from all participating patients. The results of this trial will be disseminated in one main paper and additional papers on secondary endpoints and subgroup analyses.

Trial registration number: Netherlands Trial Register (NL9525).

For peer review only - http://bmjopen.bmj.com/site/about/guidelines.xhtml

Strengths and limitations of this study (5)

- The POB-HELP study is the first randomised controlled diagnostic trial to evaluate a clinical decision rule, consisting of a risk stratification score and a high-sensitive cardiac troponin I point-of-care test (hs-cTnI-POCT), to rule out acute coronary syndrome (ACS) in patients presenting with acute chest pain in primary care.
- The clinical decision rule may reduce hospital referrals of patients with acute chest pain and, as a result, decrease the number of (unnecessary) diagnostic tests, increase patient satisfaction, relieve pressure on the healthcare system and reduce societal costs.
- The Atellica VTLi hs-cTnI analyser can provide reliable hs-cTnI measurements within only 8 minutes on several sample matrices, including the use of capillary blood obtained by a fingerstick.
- General practitioners include patients with acute chest pain based on their suspicion of ACS. Although the suspicion may differ among general practitioners (introducing inclusion bias), the included patient population will reflect daily clinical practice.
- The clinical decision rule is not applicable for patients with an onset of chest pain less than 1 hour, because troponin measurement within this time window may be false negative due to time-dependent troponin release.

For peer review only

For peer review only - http://bmjopen.bmj.com/site/about/guidelines.xhtml

Introduction

Chest pain is a common reason for consultation in primary care.(1, 2) In 4-7% of patients presenting with chest pain, the pain is caused by acute coronary syndrome (ACS).(1-3) Early identification and treatment of patients with ACS are important to avoid cardiac morbidity and mortality.(4) However, it is challenging for general practitioners (GPs) to distinguish ACS from other – less acute and life-threatening – causes of chest pain, as they have to rely on symptoms and clinical signs only.(5) Consequently, GPs refer approximately 40-70% of patients presenting with chest pain to the hospital for further examination.(1, 6)

Following referral, only 10-20% of the patients are diagnosed with ACS.(7-9) The relatively high number of referrals puts a high burden on patients and may lead to potentially avoidable adverse patient outcomes due to the risks associated with (unnecessary) diagnostic tests and overcrowding of emergency departments (ED).(10-12) In addition, a high referral rate puts a lot of pressure on the healthcare system. Finally evaluation in the ED is costly, which has a significant impact on society as well.(13) Therefore, measures to improve the referral efficiency of patients with acute chest pain is crucial.

A clinical decision rule may help GPs to safely rule out ACS and reduce hospital referrals. Previous studies in ED settings showed that a single high-sensitive cardiac troponin-I (hs-cTnI) measurement with or without a risk score is safe and effective to rule out ACS.(7, 9, 14) However, due to the low incidence of ACS in primary care, results from ED settings are not generalisable to primary care settings. Although use of a troponin point of care test (POCT) in primary care reduced the number of referrals in both a model-based and an observational study, the impact has yet to be studied in clinical practice.(15-17)

Therefore, this clustered randomised controlled diagnostic trial (RCT) aims to evaluate the impact of a primary care decision rule for acute chest pain, consisting of a risk stratification score combined with a hs-cTnI-POCT on the number of hospital referrals, and to assess its diagnostic accuracy. In addition, secondary endpoints including cost-effectiveness will be assessed.

For peer review only

For peer review only - http://bmjopen.bmj.com/site/about/guidelines.xhtml

Methods and analysis

Study design

The POB HELP study is a clustered diagnostic RCT. The study will be conducted in general practices in 3 regions in the Netherlands (Leiden, Maastricht, Venlo). General practices will be randomised to the intervention or control group in a 2:1 ratio. Randomisation is stratified for the 3 regions to ensure even distribution of rural and urban areas. Randomisation is done by Castor Electronic Data Capture using a variable block randomisation method.(18) Patient inclusion started on 18th August 2021 and is planned to end on 31st December 2023.

Study population

Patients visiting their GP, or visited by their GP, for acute chest pain symptoms in which the GP suspects an ACS, are eligible for inclusion. Exclusion criteria are: age <18 years, trauma preceding the pain, inability to speak Dutch or understand the informed consent form, onset of chest pain within less than one hour and hemodynamic instability (Table 1). Patients who match the eligibility criteria and provide informed consent are included in the study.

Table 1. In- and exclusion criteria

Inclusion	Exclusion
≥18 years of age	< 1 hour since onset of symptoms
Acute chest pain	Inability to speak or understand Dutch
	Hemodynamic instability

Regular care

In general practices randomised to the control group, included patients will receive standard care in accordance with the Dutch GP guideline for ACS.(19) Standard care consists of a medical history and physical examination by the GP. In addition, an electrocardiogram (ECG) can be performed, however an ECG device is not available in many general practices in the

For peer review only - http://bmjopen.bmj.com/site/about/guidelines.xhtml

Netherlands. Based on this information, the GP will decide to either reassure the patient ("watchful waiting"), consult a cardiologist, or refer the patient to the hospital. The GP is responsible for the follow-up of the patient.

Intervention

For patients included in general practices randomised to the intervention group, the GP will apply a clinical decision rule consisting of the Marburg Heart Score (MHS) and a hs-cTnI-POCT (Figure 1).(3, 20, 21)

The MHS consists of a 5-item score and each positive item results in 1 point: 1) Age and sex (male ≥ 55 years, female ≥ 65 years), 2) History of cardiovascular disease (i.e. stable coronary artery disease, prior myocardial infarction, coronary intervention, ischaemic cerebrovascular accident or peripheral atherosclerosis), 3) Patient assumes a cardiac origin of the chest pain, 4) Pain worsening with exercise and 5) Pain not reproducible with palpation. The ACS risk is considered low for patients with a MHS of ≤ 2 points and high for patients with ≥ 3 points.(3, 20)

The hs-cTnI measurement is performed using the Siemens Atellica VTLi immunoassay analyser.(21) Blood is obtained by a fingerstick. The droplet of blood is applied to a cartridge using a capillary transfer device. The turn-around time from the application of the droplet of blood to the test result is approximately 8 minutes. The limit of detection is 1.6 ng/L and the limit of quantitation is 8.9 ng/L (at 10% CV) and 3.7 ng/L (at 20% CV).(22) The 99th percentile overall, for men, and for women are respectively 23, 27 and 18 ng/L. A hs-cTnI level ≤ 3.8 ng/L is considered very low and is used as a cut-off to exclude ACS.(23)

The combination of the MHS and the hs-cTnI-POCT measurement results in 3 possible recommendations (Figure 1):

For peer review only

For peer review only - http://bmjopen.bmj.com/site/about/guidelines.xhtml

1) MHS ≤ 2 points and hs-cTnI-POCT ≤ 3.8 ng/L: ACS is safely excluded and watchful waiting is recommended

2) either MHS ≤ 2 points and hs-cTnI-POCT ≥ 3.9 ng/L, or MHS ≥ 3 and hs-cTnI-POCT ≤ 3.8 ng/L: ACS cannot be excluded, consultation with a cardiologist is recommended

3) MHS ≥ 3 and hs-cTnI-POCT ≥ 3.9 ng/L: The risk for ACS is increased and direct referral to the ED is warranted

If the GP or consulting cardiologist strongly disagrees with the recommendation provided by the clinical decision rule, they are allowed to overrule it. The GP is responsible for the patient's follow-up, in line with regular care.

Primary endpoints

The study has two primary endpoints. The first primary endpoint is the hospital referral rate for acute chest pain within 24 hours and 6 weeks after inclusion. The second primary endpoint is the diagnostic accuracy of the clinical decision rule. To study the diagnostic accuracy (i.e. sensitivity, negative predictive value), the incidence of ACS and major adverse cardiac events (MACE) within 6 weeks and 6 months after inclusion will be assessed. MACE is defined as a combined endpoint of ACS, percutaneous coronary intervention, coronary artery bypass grafting, coronary angiography revealing procedurally correctable stenosis managed conservatively and all-cause mortality. (7, 24, 25)

Final diagnosis

The final diagnosis will be made based on GP's medical records, including all letters from hospitals. All cases will be reviewed by an independent endpoint committee consisting of a cardiologist and general practitioner for adjudication of the final diagnosis and the occurrence of MACE. The committee will be blinded for the randomisation and hs-cTnI on presentation. In case of disagreement, a second cardiologist will be consulted. The final diagnosis will be classified as ACS, stable coronary artery disease, cardiac but non coronary disease, non-cardiac chest pain or death. ACS comprises myocardial infarction (ST-segment elevation

For peer review only - http://bmjopen.bmj.com/site/about/guidelines.xhtml

myocardial infarction (STEMI) and non-ST-segment elevation myocardial infarction (NSTEMI)) and unstable angina pectoris. Myocardial infarction is defined conform the fourth universal definition of myocardial infarction.(26)

Secondary endpoints

Secondary endpoints are the cost-effectiveness of the clinical decision rule, adherence to the recommendations of the clinical decision rule by GPs, the patient's reassurance, the diagnostic accuracy of the GP's gut feeling and the diagnostic accuracy of the HEART-score for all patients with an ECG (Table 2).(24) Finally, subgroup analyses for the primary endpoints will be performed for sex, region, socio-economic status and duration of symptoms.

Table 2. Secondary outcome measures

1.	Cost-effectiveness, consisting of a trial-based cost analysis and a cost-utility analysis (costs per QALY, assessed using the EQ-5D-5L questionnaire).(27, 28)
2.	Adherence to the recommendations of the clinical decision rule by the GP, by comparing the GP's policy with the recommendations of the decision rule.
3.	Reassurance of patients, using the State-Trait Anxiety Inventory after the index consultation.(29)
4.	Diagnostic accuracy of the GP's gut feeling, by comparing the result of the gut feeling questionnaire with the occurrence of MACE within 6 weeks after the index consultation.(30, 31)
5.	Diagnostic accuracy of the HEART-score for all patient with an ECG available for the occurrence of MACE within 6 weeks after the index consultation.(24)
6.	Subgroup analysis for sex, region, socio-economic status (using postal-code) and duration of symptoms.

For peer review only

The cost-effectiveness of the clinical decision rule will be assessed alongside the trial. The analysis will be performed from a societal perspective. The costs are divided into healthcare costs and non-healthcare costs. Healthcare costs include those of the intervention and other healthcare use during follow-up. The costs of the clinical decision rule will be based on micro-costing including the duration of the GP consultation, medical equipment and materials used. Healthcare use will be assessed by questionnaires at 6 weeks and 6 months follow-up. In this questionnaires, patients will be asked to fill out their total health care utilisation, such as GP visits, outpatient clinic visits, ED visits and hospital admissions for any reason. Reference prices published in the Dutch costing manual will be used to value healthcare use.(32) To calculate non-healthcare costs, patients will be asked to note absence from paid and unpaid work. Productivity costs will be calculated using the friction cost method. QALYs will be calculated from utility scores from the EQ-5D-5L questionnaire administered at baseline, 6 weeks and 6 months follow-up.(27, 28) Finally, costs and QALYs between the control and intervention groups will be compared.

To assess the GP's adherence to the recommendations of the decision rule, the outcome of the decision rule will be compared with the GP's policy as reported by the GP. To assess the effect of the clinical decision rule on patients' reassurance, patients will be asked to fill out the State-Trait Anxiety Inventory after the index consultation.(29) To evaluate the diagnostic accuracy of the GP's gut feeling, GPs will be asked to estimate the likelihood of ACS using the gut feeling questionnaire directly after the index consultation.(30, 31) The outcome of this questionnaire will be compared to the occurrence of MACE within 6 weeks. In all patients with an ECG, the HEART-score will be scored retrospectively based on the information on the inclusion form filled out by the GP. The outcome will be compared to the occurrence of MACE within 6 weeks.(24)

Sample size calculation

For peer review only - http://bmjopen.bmj.com/site/about/guidelines.xhtml

In primary care in the Netherlands, the reported hospital referral rate of patients with chest pain ranges from approximately 40 to 70%.(1, 6) Similarly, a Swiss and Swedish study found referral and diagnostic testing rates of 59% and 43%, respectively.(33, 34) Based on these referral rates, we conservatively assumed a hospital referral rate of 40%. For the primary endpoint, we estimated a 10% decrease in referrals, resulting in 30% referrals, which is in line with a previous study estimating a 10% decrease in referrals when using the MHS.(34, 35) Group sample sizes of 448 (control group) and 896 (intervention group) achieve 80% power to detect the difference of 10% between both groups with a significance level (alpha) of 0.05 using a two-sided Z-test.(36) The sample size is adjusted for a clustering effect with an intra-class correlation coefficient of 0.05 and for 1:2 randomisation.(37) The estimated sample size needed is 500 for the control group and 1000 for the intervention group, accounting for a 10% loss to follow-up.

Statistical analysis

Data will be analysed according to the intention-to-treat principle. Continuous demographic and baseline characteristics will be expressed as mean ± standard deviation and compared using the unpaired Student's t-test. Categorical demographic and baseline characteristics will be described as frequencies and percentages and compared using the X^2 or Fisher's exact test. The number of referrals will be presented as percentages and compared using the X^2 or Fisher's exact test. To assess the diagnostic accuracy of the decision rule, sensitivity, specificity, and predictive values will be calculated. The overall diagnostic accuracy will be assessed by means of the C-statistics. Missingness will be assessed, and if the percentage of missingness is >10%, multiple imputation will be applied. All analyses will be corrected for the correlation within clusters (general practices), using generalised estimating equations. Subgroup analyses will be performed for sex, region, socio-economic status, and duration of symptoms to confirm the applicability of the decision rule for different subgroups.

Patient involvement

For peer review only - http://bmjopen.bmj.com/site/about/guidelines.xhtml

During the design of the study, the writing of the protocol and the development of patient information materials, a participant of 'Harteraad', a patient advisory council for heart disease, was involved.

Ethical considerations

The study complies with the Declaration of Helsinki. The medical ethics committee Leiden-Den Haag-Delft has approved the protocol. Written informed consent will be obtained from all participating patients. The trial is registered in the Netherlands Trial Register (https://www.trialregister.nl/), NL9525. Accepted on 20-06-2021. A data safety monitoring board (DSMB) has been installed evaluating the safety of the study after 100, 500 and 1000 inclusions.

Discussion

This paper presents the rationale and design of a clustered diagnostic RCT in primary care. In this RCT we will evaluate the effect on the hospital referral rate, and assess the diagnostic accuracy of a primary care decision rule for patients with acute chest pain, consisting of the MHS combined with a hs-cTnI-POCT. In addition, secondary endpoints will be assessed, including the cost-effectiveness of the decision rule.

Clinical decision rule to rule out ACS in primary care

In the Netherlands, GPs handle a conservatively estimated total of 525,000 consultations for chest pain every year, and 40-70% of these patients are referred to the hospital.(1, 2, 6, 34) A clinical decision rule may support GPs to rule out ACS in primary care and avoid hospital referrals. However, in primary care settings, high-sensitive troponin measurements were unavailable until recently, and risk stratification scores without troponin measurement were found not accurate enough to rule out ACS (sensitivity 75%-91%).(8, 38) Adding a hs-cTnI-

For peer review only - http://bmjopen.bmj.com/site/about/guidelines.xhtml

POCT measurement may increase the sensitivity of a clinical decision rule in primary care to rule out ACS.

The clinical decision rule for acute chest pain in the current RCT consists of two components. The first component is the MHS, which has shown good diagnostic accuracy in ruling out stable coronary artery disease in primary care, but was insufficient to rule out ACS safely.(3, 8, 20, 38) The second component of the decision rule is a capillary hs-cTnI-POCT measurement. We hypothesise that adding this hs-cTnI-POCT to the MHS will result in a safe and accurate clinical decision rule to rule out ACS in primary care. Even though the aim of the clinical decision rule is to rule out ACS in patients with acute chest pain, it may also reduce missed ACS diagnosis in patients presenting with atypical symptoms.

Clinical implications and cost-effectiveness

In the Netherlands, GPs are the ‘gatekeeper’ to the hospital. Therefore, a clinical decision rule for acute chest pain is probably most beneficial in primary care. Based on previous literature, the clinical decision rule presented in this study may decrease hospital referrals of patients with acute chest pain by 10%.(34, 35) Thereby, this strategy may reduce uncertainty for patients and doctors, increase patient satisfaction, decrease (unnecessary) diagnostic tests and relieve pressure on healthcare personnel and the healthcare system. Furthermore, it may lead to a substantial reduction in societal costs as was shown in a previous observational Norwegian study.(17) In the Netherlands, a patient referred to the hospital for acute chest pain on average costs €1580, whereas a primary care consultation plus hs-cTnI-POCT measurement is estimated to cost on average €73.(13, 32) Therefore, if the decision rule would be implemented in 50% of general practices and assuming a 10% reduction in referrals, this could potentially lead to an estimated cost-reduction of €31 million in The Netherlands each year.(39)

Generalisability

For peer review only - http://bmjopen.bmj.com/site/about/guidelines.xhtml

The current RCT will compare the clinical decision rule to regular care in general practices in three regions in The Netherlands, including urban and rural areas. After careful instruction on the inclusion criteria, the GPs in these practices will decide on the eligibility of individual patients. Although differences among GPs (e.g. a less experienced GP may include different patients than an experienced GP) may lead to inclusion bias, this situation resembles daily clinical practice. Hence, the results of this study will be generalisable to the rest of The Netherlands and other countries with a similar primary care system, where the GP functions as a gatekeeper (e.g. the UK, Scandinavian countries and Canada). Additionally, we will measure the GP's working experience, which enables us to quantify this potential bias.

Strengths and limitations

The present study is a diagnostic RCT in primary care, with sufficient power to evaluate a significant difference in the hospital referral rate when applying the clinical decision rule compared to regular care. To the best of our knowledge, this is the first RCT studying the clinical application of a decision rule for acute chest pain, including a hs-cTnI-POCT in primary care.

A major strength of the study is the use of the Atellica VTLi hs-cTnI analyser which can provide reliable hs-cTnI measurements within approximately 8 minutes on several sample matrices, including the use of capillary blood obtained by a fingerstick.(21, 23, 40) The use of a fingerstick makes it highly suitable for a pre-hospital setting. Point of care testing has the potential to add great value to the evaluation of patients with chest pain in primary care. This is, in part, because neither sample transport nor centrifugation of the sample is necessary if a whole blood sample is tested.

The study has three main limitations. First, the diagnostic accuracy of the decision rule cannot be assessed using the golden standard for ACS patients. A coronary angiogram (CAG) is the golden standard to establish the presence of coronary artery disease. However, CAG is costly

For peer review only - http://bmjopen.bmj.com/site/about/guidelines.xhtml

and not without risk (e.g. bleeding in approximately 7% and procedure-related myocardial infarction in approximately 6% of patients undergoing CAG).(41, 42) Therefore, we considered it too invasive and essentially unethical to perform a CAG in each patient in this relatively low-risk population. Hence, we will use a delayed reference standard, defined as ACS and MACE within 6 weeks and 6 months after the index consultation, to determine the diagnostic accuracy of the decision rule. The delayed reference method has been reported as a valid alternative if a golden standard test is too invasive and the disease is not self-limiting.(43) This method has been used in similar studies before.(7, 20, 25, 44) The second limitation is that the decision rule is not applicable for patients with an onset of chest pain less than 1 hour, due to the time-depended release of troponin, which may result in false negative troponin measurements within this period. However, this is only 10% of all patients presenting with acute chest pain.(8, 45) Finally, an ECG is not included in the decision rule, as an ECG device is not available in many general practices in The Netherlands. Nonetheless, GPs with an ECG device available are free to make an ECG and overrule the recommendations of the clinical decision rule as they deem necessary. However, it is important to notice, that an ECG may be normal in 30% of patients presenting with non-ST elevated myocardial infarction and is therefore unsuitable for excluding ACS safely.(46)

Conclusion

In summary, this manuscript presents the rationale and design of a diagnostic RCT to rule out ACS in primary care using a clinical decision rule consisting of the MHS and a hs-cTnI-POCT. In this study, we will evaluate the impact of this decision rule on hospital referrals and assess its diagnostic accuracy. Additionally, the cost-effectiveness will be assessed. If found to be safe and effective the clinical decision rule will strengthen the position of the GP as gatekeeper and reduce referrals of patients with acute chest pain to secondary care, hence diminishing pressure on the healthcare system, reducing societal costs and increase patient satisfaction.

For peer review only

Author's contributions

SB, TB, RW, MN and GD have written the protocol of the study. MB, JM and BR substantially revised the protocol. MA has written the section on cost-effectiveness analyses. SB and AP have written this manuscript. All authors read and substantially revised the manuscript.

Funding

This work was supported by ZonMw (project ID 852001907). The Atellica VTLi immunoassay analysers used for the hs-cTnI-POCT measurements in this study are provided by Siemens Healthineers.

Competing interest statement

The authors have no conflicts of interest to declare. Siemens Healthineers provides the immunoassay analysers, cartridges and quality control samples. However, Siemens Healthineers had no role in the design of the study and the results will be analysed and published by the independent researchers.

For peer review only

For peer review only - http://bmjopen.bmj.com/site/about/guidelines.xhtml

References

1. Hoorweg BBN, Willemsen RTA, Cleef L, et al. Frequency of chest pain in primary care, diagnostic tests performed and final diagnoses. Heart. 2017;103:1727-32.

2. Bosner S, Becker A, Haasenritter J, et al. Chest pain in primary care: epidemiology and pre-work-up probabilities. Eur J Gen Pract. 2009;15:141-6.

3. Bosner S, Haasenritter J, Becker A, et al. Ruling out coronary artery disease in primary care: development and validation of a simple prediction rule. CMAJ. 2010;182:1295-300.

4. Terkelsen CJ, Sørensen JT, Maeng M, et al. System delay and mortality among patients with STEMI treated with primary percutaneous coronary intervention. Jama. 2010;304(7):763-71.

5. Harskamp R, van Peet P, Bont J, et al. The conundrum of acute chest pain in general practice: a nationwide survey in The Netherlands. BJGP Open. 2018;2:1-10.

6. Bruins Slot MH, Rutten FH, van der Heijden GJ, et al. Diagnosing acute coronary syndrome in primary care: comparison of the physicians' risk estimation and a clinical decision rule. Fam Pract. 2011;28(3):323-8.

7. Poldervaart JM, Reitsma JB, Backus BE, et al. Effect of Using the HEART Score in Patients With Chest Pain in the Emergency Department. Annals of Internal Medicine. 2017;166(10):689.

8. Schols AMR, Willemsen RTA, Bonten TN, et al. A Nationwide Flash-Mob Study for Suspected Acute Coronary Syndrome. The Annals of Family Medicine. 2019;17(4):296-303.

9. Chiang CH, Chiang CH, Pickering JW, et al. Performance of the European Society of Cardiology 0/1-Hour, 0/2-Hour, and 0/3-Hour Algorithms for Rapid Triage of Acute Myocardial Infarction : An International Collaborative Meta-analysis. Ann Intern Med. 2022;175(1):101-13.

10. Pines JM, Pollack CV, Jr., Diercks DB, et al. The association between emergency department crowding and adverse cardiovascular outcomes in patients with chest pain. Acad Emerg Med. 2009;16(7):617-25.

For peer review only - http://bmjopen.bmj.com/site/about/guidelines.xhtml

370 11. Sun BC, Hsia RY, Weiss RE, et al. Effect of emergency department crowding on
371 outcomes of admitted patients. Ann Emerg Med. 2013;61(6):605-11 e6.
372 12. Rasouli HR, Esfahani AA, Nobakht M, et al. Outcomes of Crowding in Emergency
373 Departments; a Systematic Review. Arch Acad Emerg Med. 2019;7(1):e52.
374 13. van Dongen DN, Ottervanger JP, Tolsma R, et al. In-Hospital Healthcare Utilization,
375 Outcomes, and Costs in Pre-Hospital-Adjudicated Low-Risk Chest-Pain Patients. Appl
376 Health Econ Health Policy. 2019;17(6):875-82.
377 14. Pickering JW, Than MP, Cullen L, et al. Rapid Rule-out of Acute Myocardial
378 Infarction With a Single High-Sensitivity Cardiac Troponin T Measurement Below the Limit of
379 Detection: A Collaborative Meta-analysis. Annals of Internal Medicine. 2017;166(10):715-24.
380 15. Kip MMA, Koffijberg H, Moesker MJ, et al. The cost-utility of point-of-care troponin
381 testing to diagnose acute coronary syndrome in primary care. BMC Cardiovasc Disord.
382 2017;17(1):213.
383 16. Johannessen TR, Vallersnes OM, Halvorsen S, et al. Pre-hospital One-Hour
384 Troponin in a Low-Prevalence Population of Acute Coronary Syndrome: OUT-ACS study.
385 Open Heart. 2020;7(2).
386 17. Johannessen TR, Halvorsen S, Atar D, et al. Cost-effectiveness of a rule-out
387 algorithm of acute myocardial infarction in low-risk patients: emergency primary care versus
388 hospital setting. BMC Health Serv Res. 2022;22(1):1274.
389 18. Castor EDC. Castor Electronic Data Capture 2019 [online]. 2019. Available from:
390 https://castoredc.com.(accessed 14 December 2022)
391 19. Auwerda A, Bouma M, Bruins Slot M, et al. NHG standaard Acuut coronair syndroom
392 2022 [online]. 2022. Available from: https://richtlijnen.nhg.org/standaarden/acuut-coronair-
393 syndroom.(accessed 14 December 2022)
394 20. Haasenritter J, Bosner S, Vaucher P, et al. Ruling out coronary heart disease in
395 primary care: external validation of a clinical prediction rule. The British journal of general
396 practice : the journal of the Royal College of General Practitioners. 2012;62:415-21.

For peer review only - http://bmjopen.bmj.com/site/about/guidelines.xhtml

21. Apple FS, Schulz K, Schmidt CW, et al. Determination of sex-specific 99th percentile upper reference limits for a point of care high sensitivity cardiac troponin I assay. Clin Chem Lab Med. 2021;59(9):1574-8.

22. Christenson RH, Frenk LDS, de Graaf HJ, et al. Point-of-Care: Roadmap for Analytical Characterization and Validation of a High-Sensitivity Cardiac Troponin I Assay in Plasma and Whole Blood Matrices. J Appl Lab Med. 2022;7(4):971-88.

23. Apple FS, Smith SW, Greenslade JH, et al. Single High-Sensitivity Point of Care Whole Blood Cardiac Troponin I Measurement to Rule Out Acute Myocardial Infarction at Low Risk. Circulation. 2022.

24. Backus BE, Six AJ, Kelder JC, et al. A prospective validation of the HEART score for chest pain patients at the emergency department. International Journal of Cardiology. 2013;168(3):2153-8.

25. van Dongen DN, Fokkert MJ, Tolsma RT, et al. Accuracy of pre-hospital HEART score risk classification using point of care versus high sensitive troponin in suspected NSTE-ACS. Am J Emerg Med. 2020;38(8):1616-20.

26. Thygesen K, Alpert JS, Jaffe AS, et al. Fourth universal definition of myocardial infarction (2018). Eur Heart J. 2019;40(3):237-69.

27. Herdman M, Gudex C, Lloyd A, et al. Development and preliminary testing of the new five-level version of EQ-5D (EQ-5D-5L). Qual Life Res. 2011;20(10):1727-36.

28. Versteegh MM, Vermeulen KM, Evers SMAA, et al. Dutch Tariff for the Five-Level Version of EQ-5D. Value Health. 2016;19(4):343-52.

29. Spielberger CD. State-trait anxiety inventory for adults. 1983.

30. Stolper CF, Van de Wiel MWJ, De Vet HCW, et al. Family physicians' diagnostic gut feelings are measurable: construct validation of a questionnaire. BMC Family practice. 2013;14(1).

31. Barais M, Fossard E, Dany A, et al. Accuracy of the general practitioner's sense of alarm when confronted with dyspnoea and/or chest pain: a prospective observational study. BMJ Open. 2020;10(2):e034348.

For peer review only

For peer review only - http://bmjopen.bmj.com/site/about/guidelines.xhtml

32. Kanters TA, Bouwmans CAM, van der Linden N, et al. Update of the Dutch manual for costing studies in health care. PLoS One. 2017;12(11):e0187477.

33. Verdon F, Herzig L, Burnand B, et al. Chest pain in daily practice: occurrence, causes and management. Swiss med wkly. 2008;138:340-7.

34. Nilsson S, Andersson A, Janzon M, et al. Cost consequences of point-of-care troponin T testing in a Swedish primary health care setting. Scand J Prim Health Care. 2014;32(4):241-7.

35. Haasenritter J, Donner-Banzhoff N, Bösner S. Chest pain for coronary heart disease in general practice: clinical judgement and a clinical decision rule. The British journal of general practice : the journal of the Royal College of General Practitioners. 2015.

36. Donner A, Klar N. Design and Analysis of Cluster Randomization Trials in Health Research. London: Arnold Publishers; 2000.

37. Smeeth L, Ng ES-W. Intraclass correlation coefficients for cluster randomized trials in primary care: data from the MRC Trial of the Assessment and management of Older People in the Community. Control Clin Trials. 2002;23:409-21.

38. Harskamp R, Laeven SC, Himmelreich J, et al. Chest pain in general practice: a systematic review of prediction rules. BMJ open. 2018;9:1-10.

39. Grol R, Dalhuijsen J, Thomas S, et al. Attributes of clinical guidelines that influence use of guidelines in general practice: observational study. Bmj. 1998;317(7162):858-61.

40. Koper LH, Frenk LDS, Meeder JG, et al. URGENT 1.5: diagnostic accuracy of the modified HEART score, with fingerstick point-of-care troponin testing, in ruling out acute coronary syndrome. Neth Heart J. 2022;30(7-8):360-9.

41. Mehta SR, Cannon CP, Fox KA, et al. Routine vs selective invasive strategies in patients with acute coronary syndromes: a collaborative meta-analysis of randomized trials. Jama. 2005;293(23):2908-17.

42. Fanning JP, Nyong J, Scott IA, et al. Routine invasive strategies versus selective invasive strategies for unstable angina and non-ST elevation myocardial infarction in the stent era. Cochrane Database Syst Rev. 2016;2016(5):Cd004815.

For peer review only

For peer review only - http://bmjopen.bmj.com/site/about/guidelines.xhtml

43. Knottnerus JA, Muris JW. Assessment of the accuracy of diagnostic tests: the cross-sectional study. Journal of Clinical Epidemiology. 2003;56(11):1118-28.

44. Tolsma RT, Fokkert MJ, van Dongen DN, et al. Referral decisions based on a pre-hospital HEART score in suspected non-ST-elevation acute coronary syndrome: final results of the FamouS Triage study. Eur Heart J Acute Cardiovasc Care. 2021.

45. Andersen CF, Bang C, Lauridsen KG, et al. Single troponin measurement to rule-out acute myocardial infarction in early presenters. Int J Cardiol. 2021.

46. Collet JP, Thiele H, Barbato E, et al. 2020 ESC Guidelines for the management of acute coronary syndromes in patients presenting without persistent ST-segment elevation. Eur Heart J. 2020.

For peer review only

463 Figure 1. Flowchart of the clinical decision rule

For peer review only

For peer review only - http://bmjopen.bmj.com/site/about/guidelines.xhtml

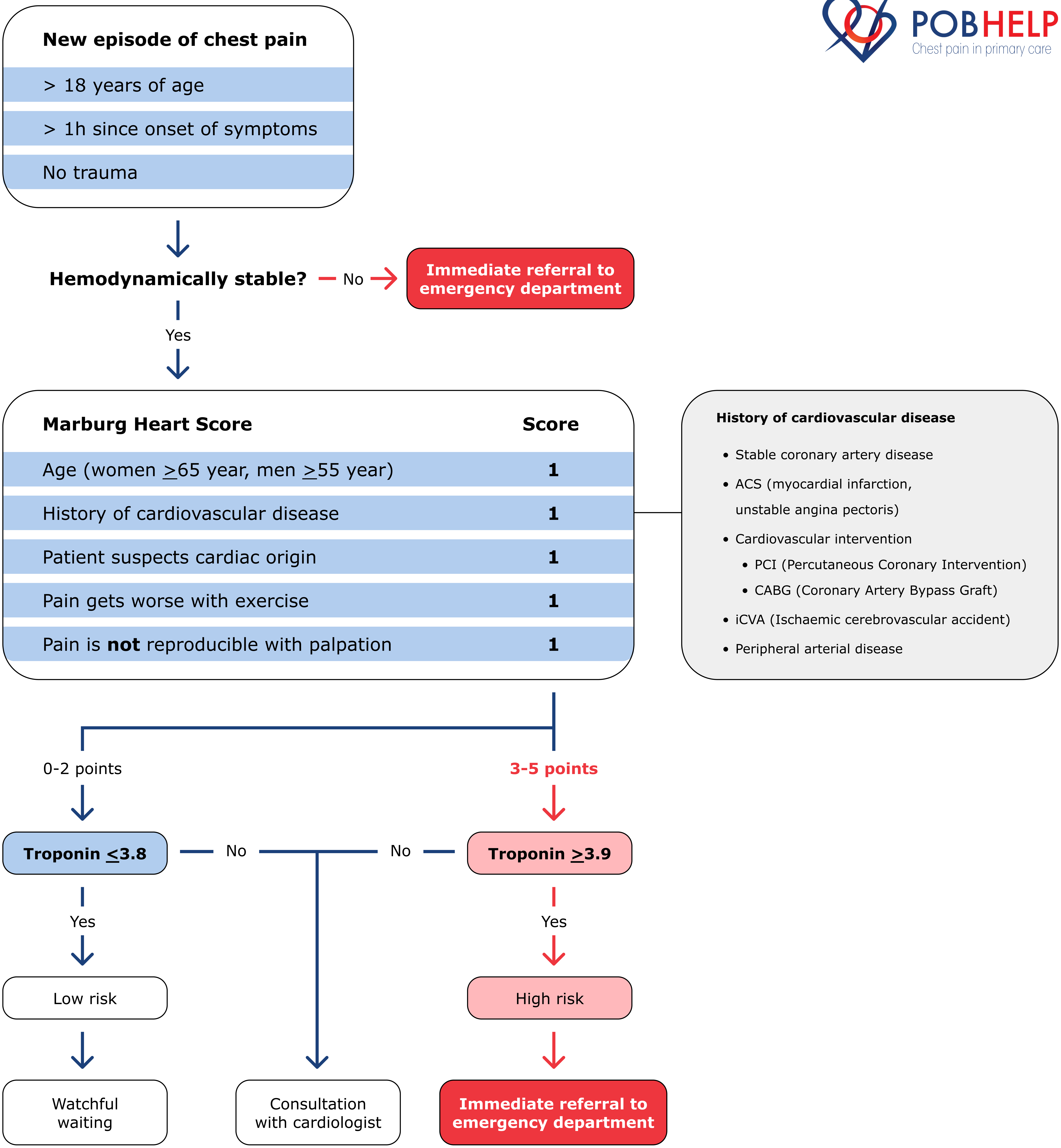
POBHELP
Chest pain in primary care
New episode of chest pain
> 18 years of age
> 1h since onset of symptoms
No trauma
Hemodynamically stable?
No
Immediate referral to emergency department
Yes
Marburg Heart Score
Score
Age (women >65 year, men >55 year) 1
History of cardiovascular disease 1
Patient suspects cardiac origin 1
Pain gets worse with exercise 1
Pain is not reproducible with palpation 1
History of cardiovascular disease
Stable coronary artery disease
ACS (myocardial infarction, unstable angina pectoris)
Cardiovascular intervention
PCI (Percutaneous Coronary Intervention)
CABG (Coronary Artery Bypass Graft)
iCVA (Ischaemic cerebrovascular accident)
Peripheral arterial disease
0-2 points
3-5 points
Troponin <3.8
No
No
Troponin >3.9
Yes
Yes
Low risk
High risk
Watchful waiting
Consultation with cardiologist
Immediate referral to emergency department

SPIRIT

STANDARD PROTOCOL ITEMS: RECOMMENDATIONS FOR INTERVENTIONAL TRIALS

SPIRIT 2013 Checklist: Recommended items to address in a clinical trial protocol and related documents*

Section/item	ItemNo	Description	
**Administrative information**			
Title	1	Descriptive title identifying the study design, population, interventions, and, if applicable, trial acronym	x
Trial registration	2a	Trial identifier and registry name. If not yet registered, name of intended registry	x
	2b	All items from the World Health Organization Trial Registration Data Set	x
Protocol version	3	Date and version identifier	x
Funding	4	Sources and types of financial, material, and other support	x
Roles and responsibilities	5a	Names, affiliations, and roles of protocol contributors	x
	5b	Name and contact information for the trial sponsor	x
	5c	Role of study sponsor and funders, if any, in study design; collection, management, analysis, and interpretation of data; writing of the report; and the decision to submit the report for publication, including whether they will have ultimate authority over any of these activities	x
	5d	Composition, roles, and responsibilities of the coordinating centre, steering committee, endpoint adjudication committee, data management team, and other individuals or groups overseeing the trial, if applicable (see Item 21a for data monitoring committee)	
**Introduction**			
Background and rationale	6a	Description of research question and justification for undertaking the trial, including summary of relevant studies (published and unpublished) examining benefits and harms for each intervention	x
	6b	Explanation for choice of comparators	x
Objectives	7	Specific objectives or hypotheses	x

For peer review only

Trial design	8	Description of trial design including type of trial (eg, parallel group, crossover, factorial, single group), allocation ratio, and framework (eg, superiority, equivalence, noninferiority, exploratory)	x
**Methods: Participants, interventions, and outcomes**			
Study setting	9	Description of study settings (eg, community clinic, academic hospital) and list of countries where data will be collected. Reference to where list of study sites can be obtained	x
Eligibility criteria	10	Inclusion and exclusion criteria for participants. If applicable, eligibility criteria for study centres and individuals who will perform the interventions (eg, surgeons, psychotherapists)	x
Interventions	11a	Interventions for each group with sufficient detail to allow replication, including how and when they will be administered	x
	11b	Criteria for discontinuing or modifying allocated interventions for a given trial participant (eg, drug dose change in response to harms, participant request, or improving/worsening disease)	n/a
	11c	Strategies to improve adherence to intervention protocols, and any procedures for monitoring adherence (eg, drug tablet return, laboratory tests)	n/a
	11d	Relevant concomitant care and interventions that are permitted or prohibited during the trial	n/a
Outcomes	12	Primary, secondary, and other outcomes, including the specific measurement variable (eg, systolic blood pressure), analysis metric (eg, change from baseline, final value, time to event), method of aggregation (eg, median, proportion), and time point for each outcome. Explanation of the clinical relevance of chosen efficacy and harm outcomes is strongly recommended	x
Participant timeline	13	Time schedule of enrolment, interventions (including any run-ins and washouts), assessments, and visits for participants. A schematic diagram is highly recommended (see Figure)	x
Sample size	14	Estimated number of participants needed to achieve study objectives and how it was determined, including clinical and statistical assumptions supporting any sample size calculations	x
Recruitment	15	Strategies for achieving adequate participant enrolment to reach target sample size	x
**Methods: Assignment of interventions (for controlled trials)**			
Allocation:			

Sequence generation	16a	Method of generating the allocation sequence (eg, computer-generated random numbers), and list of any factors for stratification. To reduce predictability of a random sequence, details of any planned restriction (eg, blocking) should be provided in a separate document that is unavailable to those who enrol participants or assign interventions	x
Allocation concealment mechanism	16b	Mechanism of implementing the allocation sequence (eg, central telephone; sequentially numbered, opaque, sealed envelopes), describing any steps to conceal the sequence until interventions are assigned	n/a
Implementation	16c	Who will generate the allocation sequence, who will enrol participants, and who will assign participants to interventions	x
Blinding (masking)	17a	Who will be blinded after assignment to interventions (eg, trial participants, care providers, outcome assessors, data analysts), and how	n/a
	17b	If blinded, circumstances under which unblinding is permissible, and procedure for revealing a participant's allocated intervention during the trial	n/a
**Methods: Data collection, management, and analysis**			
Data collection methods	18a	Plans for assessment and collection of outcome, baseline, and other trial data, including any related processes to promote data quality (eg, duplicate measurements, training of assessors) and a description of study instruments (eg, questionnaires, laboratory tests) along with their reliability and validity, if known. Reference to where data collection forms can be found, if not in the protocol	x
	18b	Plans to promote participant retention and complete follow-up, including list of any outcome data to be collected for participants who discontinue or deviate from intervention protocols	x
Data management	19	Plans for data entry, coding, security, and storage, including any related processes to promote data quality (eg, double data entry; range checks for data values). Reference to where details of data management procedures can be found, if not in the protocol	x
Statistical methods	20a	Statistical methods for analysing primary and secondary outcomes. Reference to where other details of the statistical analysis plan can be found, if not in the protocol	x
	20b	Methods for any additional analyses (eg, subgroup and adjusted analyses)	x

For peer review only - http://bmjopen.bmj.com/site/about/guidelines.xhtml

	20c	Definition of analysis population relating to protocol non-adherence (eg, as randomised analysis), and any statistical methods to handle missing data (eg, multiple imputation)	x
**Methods: Monitoring**			
Data monitoring	21a	Composition of data monitoring committee (DMC); summary of its role and reporting structure; statement of whether it is independent from the sponsor and competing interests; and reference to where further details about its charter can be found, if not in the protocol. Alternatively, an explanation of why a DMC is not needed	x
	21b	Description of any interim analyses and stopping guidelines, including who will have access to these interim results and make the final decision to terminate the trial	n/a
Harms	22	Plans for collecting, assessing, reporting, and managing solicited and spontaneously reported adverse events and other unintended effects of trial interventions or trial conduct	x
Auditing	23	Frequency and procedures for auditing trial conduct, if any, and whether the process will be independent from investigators and the sponsor	x
**Ethics and dissemination**			
Research ethics approval	24	Plans for seeking research ethics committee/institutional review board (REC/IRB) approval	x
Protocol amendments	25	Plans for communicating important protocol modifications (eg, changes to eligibility criteria, outcomes, analyses) to relevant parties (eg, investigators, REC/IRBs, trial participants, trial registries, journals, regulators)	x
Consent or assent	26a	Who will obtain informed consent or assent from potential trial participants or authorised surrogates, and how (see Item 32)	x
	26b	Additional consent provisions for collection and use of participant data and biological specimens in ancillary studies, if applicable	x
Confidentiality	27	How personal information about potential and enrolled participants will be collected, shared, and maintained in order to protect confidentiality before, during, and after the trial	x
Declaration of interests	28	Financial and other competing interests for principal investigators for the overall trial and each study site	n/a
Access to data	29	Statement of who will have access to the final trial dataset, and disclosure of contractual agreements that limit such access for investigators	x

review only

Ancillary and post-trial care	30	Provisions, if any, for ancillary and post-trial care, and for compensation to those who suffer harm from trial participation	x
Dissemination policy	31a	Plans for investigators and sponsor to communicate trial results to participants, healthcare professionals, the public, and other relevant groups (eg, via publication, reporting in results databases, or other data sharing arrangements), including any publication restrictions	x
	31b	Authorship eligibility guidelines and any intended use of professional writers	-
	31c	Plans, if any, for granting public access to the full protocol, participant-level dataset, and statistical code	-
**Appendices**			
Informed consent materials	32	Model consent form and other related documentation given to participants and authorised surrogates	x
Biological specimens	33	Plans for collection, laboratory evaluation, and storage of biological specimens for genetic or molecular analysis in the current trial and for future use in ancillary studies, if applicable	n/a

*It is strongly recommended that this checklist be read in conjunction with the SPIRIT 2013 Explanation & Elaboration for important clarification on the items. Amendments to the protocol should be tracked and dated. The SPIRIT checklist is copyrighted by the SPIRIT Group under the Creative Commons "Attribution-NonCommercial-NoDerivs 3.0 Unported" license.

For peer review only - http://bmjopen.bmj.com/site/about/guidelines.xhtml

BMJ Open

Ruling out acute coronary syndrome in primary care with a clinical decision rule and a capillary, high-sensitive troponin I point of care test: Study protocol of a diagnostic RCT in the Netherlands (POB HELP)

Journal:	*BMJ Open*
Manuscript ID	bmjopen-2023-071822.R1
Article Type:	Protocol
Date Submitted by the Author:	02-May-2023
Complete List of Authors:	van den Bulk, Simone; Leiden Universitair Medisch Centrum, Public Health and primary Care<br>Petrus, Annelieke; Leiden Universitair Medisch Centrum, Public Health and primary Care<br>Willemsen, Robert ; Maastricht University, Department of Family Medicine<br>Boogers, Mark; Leiden Universitair Medisch Centrum, Cardiology<br>Meeder, Joan; VieCuri Medisch Centrum voor Noord-Limburg, Cardiology<br>Rahel, Braim; VieCuri Medisch Centrum voor Noord-Limburg, Cardiology<br>van den Akker-van Marle, Elske; Leiden University Medical Center, Biomedical Data Sciences<br>Numans, Mattijs; Leiden Universitair Medisch Centrum, Public Health and primary Care<br>Dinant, Geert-Jan; Maastricht University, Department of Family Medicine<br>Bonten, Tobias N.; Leids Universitair Medisch Centrum, Public Health and primary Care
**Primary Subject Heading**:	General practice / Family practice
Secondary Subject Heading:	Cardiovascular medicine, Diagnostics
Keywords:	Myocardial infarction < CARDIOLOGY, PRIMARY CARE, Decision Making, ACCIDENT & EMERGENCY MEDICINE

SCHOLARONE™
Manuscripts

Ruling out acute coronary syndrome in primary care with a clinical decision rule and a capillary, high-sensitive troponin I point of care test: Study protocol of a diagnostic RCT in the Netherlands (POB HELP)

S. van den Bulk MD[1], A.H.J. Petrus MD PhD[1], R.T. Willemsen MD PhD[2], M.J. Boogers[3], J.G. Meeder[4], B.M. Rahel[4], M.E. van den Akker-van Marle[5], PhD, M.E. Numans MD PhD[1], G.J. Dinant MD PhD[2], T.N. Bonten MD PhD[1]

Word count: 3598

Addresses of authors

1 Department of Public Health and Primary Care
Leiden University Medical Center
Post zone V0-P
Postbus 9600
2300 RC Leiden, The Netherlands

2 Faculty of Health, Medicine and Life Sciences
Maastricht University Medical Center+

PO BOX 616
6200 MD Maastricht, The Netherlands

3 Department of Cardiology
Leiden University Medical Center
Post zone V0-P
Postbus 9600
2300 RC Leiden, The Netherlands

4 Department of Cardiology
VieCuri Medical center for Noord-Limburg
PO BOX 1926
5900 BX Venlo, The Netherlands

5 Department of Biomedical Data Sciences,
unit Medical Decision Making
Leiden University Medical Center
Post zone J10-S
Postbus 9600
2300 RC Leiden, The Netherlands

Corresponding author

Simone van den Bulk, MD
@ s.van_den_bulk@lumc.nl
T: +31 625043440

Department of Public Health and Primary Care
Leiden University Medical Center
Post zone V0-P
Postbus 9600
2300 RC Leiden, The Netherland

For peer review only - http://bmjopen.bmj.com/site/about/guidelines.xhtml

Abstract

Introduction: Chest pain is a common reason for consultation in primary care. To rule out acute coronary syndrome (ACS), general practitioners (GP) refer 40-70% of patients with chest pain to the emergency department (ED). Only 10-20% of those referred, are diagnosed with ACS. A clinical decision rule, including a high-sensitive cardiac troponin-I point-of-care test (hs-cTnI-POCT), may safely rule out ACS in primary care. Being able to safely rule out ACS at the GP level reduces referrals and thereby alleviates the burden on the ED. Moreover, prompt feedback to the patients may reduce anxiety and stress.

Methods and analysis: The POB HELP study is a clustered randomised controlled diagnostic trial investigating the (cost-)effectiveness and diagnostic accuracy of a primary care decision rule for acute chest pain, consisting of the Marburg Heart Score combined with a hs-cTnI-POCT (limit of detection 1.6 ng/L, 99th percentile 23 ng/L, cut-off value between negative and positive used in this study 3.8 ng/L). General practices are 2:1 randomised to the intervention group (clinical decision rule) or control group (regular care). In total 1500 patients with acute chest pain are planned to be included by GPs in three regions in The Netherlands. Primary endpoints are the number of hospital referrals and the diagnostic accuracy of the decision rule 24 hours, 6 weeks and 6 months after inclusion.

Ethics and dissemination: The medical ethics committee Leiden-Den Haag-Delft (the Netherlands) has approved this trial. Written informed consent will be obtained from all participating patients. The results of this trial will be disseminated in one main paper and additional papers on secondary endpoints and subgroup analyses.

Trial registration number: Netherlands Trial Register (NL9525) and www.clinicaltrials.gov (NCT05827237).

For peer review only - http://bmjopen.bmj.com/site/about/guidelines.xhtml

Strengths and limitations of this study (5)

- The study design is a randomised controlled diagnostic trial, which prospectively evaluates a clinical decision rule, including a high-sensitive cardiac troponin I (hs-cTnI) point-of-care test, to rule out acute coronary syndrome (ACS) in primary care.
- The study includes a cost-effectiveness analysis, to evaluate the efficiency of the clinical decision rule and the impact on the healthcare system.
- The Atellica VTLi hs-cTnI analyser provides reliable hs-cTnI measurements within only 8 minutes in capillary blood obtained by a fingerstick.
- Inclusion is based on clinical suspicion of ACS by general practitioners, which reflects daily clinical practice, but may lead to inclusion bias.
- Patients with an onset of chest pain <1 hour are excluded, because hs-cTnI measurement within this time window may be false negative due to time-dependent troponin release.

For peer review only

Introduction

Chest pain is a common reason for consultation in primary care.(1, 2) In 4-7% of patients presenting with chest pain, the pain is caused by acute coronary syndrome (ACS).(1-3) Early identification and treatment of patients with ACS are important to avoid cardiac morbidity and mortality.(4) However, it is challenging for general practitioners (GPs) to distinguish ACS from other – less acute and life-threatening – causes of chest pain, as they have to rely on symptoms and clinical signs only.(5) Consequently, GPs refer approximately 40-70% of patients presenting with chest pain to the hospital for further examination.(1, 6)

Following referral, only 10-20% of the patients are diagnosed with ACS.(7-9) The relatively high number of referrals puts a high burden on patients and may lead to potentially avoidable adverse patient outcomes due to the risks associated with (unnecessary) diagnostic tests and overcrowding of emergency departments (ED).(10-12) In addition, a high referral rate puts a lot of pressure on the healthcare system. Finally, evaluation of patients in the ED is costly, which has a significant impact on society as well.(13) Therefore, measures to improve the referral efficiency of patients with acute chest pain is crucial.

A clinical decision rule may help GPs to safely rule out ACS and reduce hospital referrals. Previous studies in ED settings showed that a single high-sensitive cardiac troponin-I (hs-cTnI) measurement with or without a risk score is safe and effective to rule out ACS.(7, 9, 14) However, due to the low incidence of ACS in primary care, results from ED settings are not generalisable to primary care settings. Although use of a troponin point of care test (POCT) in primary care reduced the number of referrals in both a model-based and an observational study, the impact has yet to be studied in clinical practice.(15-17)

Therefore, this clustered randomised controlled diagnostic trial (RCT) aims to evaluate the impact of a primary care decision rule for acute chest pain, consisting of a risk stratification score combined with a hs-cTnI-POCT on the number of hospital referrals, and to assess its diagnostic accuracy. In addition, secondary endpoints including cost-effectiveness will be assessed.

For peer review only - http://bmjopen.bmj.com/site/about/guidelines.xhtml

Methods and analysis

Study design

The POB HELP study is a clustered diagnostic RCT. The study will be conducted in general practices in 3 regions in the Netherlands (Leiden, Maastricht, Venlo). General practices will be randomised to the intervention or control group in a 2:1 ratio. Randomisation is stratified for the 3 regions to ensure even distribution of rural and urban areas. Randomisation is done by Castor Electronic Data Capture using a variable block randomisation method.(18) Patient inclusion started on 18th August 2021 and is planned to end on 31st December 2023.

Study population

Patients visiting their GP, or visited by their GP, for acute chest pain symptoms in which the GP suspects an ACS, are eligible for inclusion. Patients who contact their GP by telephone with acute chest pain highly suspicious for ACS are not eligible for inclusion. For these patients an ambulance is sent directly to the home address by the doctor's assistant, in accordance with usual care. Exclusion criteria are: age <18 years, trauma preceding the pain, inability to speak Dutch or understand the informed consent form, onset of chest pain within less than one hour and hemodynamic instability (Table 1). Patients who match the eligibility criteria and provide informed consent are included in the study.

Table 1. In- and exclusion criteria

Inclusion	Exclusion
≥18 years of age	< 1 hour since onset of symptoms
Acute chest pain	Inability to speak or understand Dutch
Seen by the general practitioner	Hemodynamic instability

Regular care

For peer review only - http://bmjopen.bmj.com/site/about/guidelines.xhtml

In general practices randomised to the control group, included patients will receive standard care in accordance with the Dutch GP guideline for ACS.(19) Standard care consists of a medical history and physical examination by the GP. In addition, an electrocardiogram (ECG) can be performed, however an ECG device is not available in many general practices in the Netherlands. Based on this information, the GP will decide to either reassure the patient ("watchful waiting"), consult a cardiologist, or refer the patient to the hospital. The GP is responsible for the follow-up of the patient.

Intervention

For patients included in general practices randomised to the intervention group, the GP will apply a clinical decision rule consisting of the Marburg Heart Score (MHS) and a hs-cTnI-POCT (Figure 1).(3, 20, 21)

The MHS consists of a 5-item score and each positive item results in 1 point: 1) Age and sex (male ≥ 55 years, female ≥ 65 years), 2) History of cardiovascular disease (i.e. stable coronary artery disease, prior myocardial infarction, coronary intervention, ischaemic cerebrovascular accident or peripheral atherosclerosis), 3) Patient assumes a cardiac origin of the chest pain, 4) Pain worsening with exercise and 5) Pain not reproducible with palpation. The ACS risk is considered low for patients with a MHS of ≤ 2 points and high for patients with ≥ 3 points.(3, 20)

The hs-cTnI measurement is performed using the Siemens Atellica VTLi immunoassay analyser.(21) The analyser is easy to use and guides the user step by step through the process. Prior to the start of the study, the staff, including the doctor's assistants and at least one GP per general practice, is trained by a study employee. A study employee is available during office hours to provide assistance by telephone when necessary. Blood is obtained by a fingerstick, which makes it highly suitable for a pre-hospital setting. The droplet of blood is applied to a cartridge using a capillary transfer device. The turn-around time from the

For peer review only - http://bmjopen.bmj.com/site/about/guidelines.xhtml

application of the droplet of blood to the test result is approximately 8 minutes. The limit of detection is 1.6 ng/L and the limit of quantitation is 8.9 ng/L (at 10% CV) and 3.7 ng/L (at 20% CV).(22) The 99th percentile overall, for men, and for women are respectively 23, 27 and 18 ng/L. A hs-cTnI level ≤ 3.8 ng/L is considered very low and is used as a cut-off in this study to exclude ACS.(23)

The combination of the MHS and the hs-cTnI-POCT measurement results in 3 possible recommendations (Figure 1):

1) MHS ≤ 2 points and hs-cTnI-POCT ≤ 3.8 ng/L: ACS is safely excluded and watchful waiting is recommended.

2) either MHS ≤ 2 points and hs-cTnI-POCT ≥ 3.9 ng/L, or MHS ≥ 3 and hs-cTnI-POCT ≤ 3.8 ng/L: ACS cannot be excluded, consultation with a cardiologist is recommended.

3) MHS ≥ 3 and hs-cTnI-POCT ≥ 3.9 ng/L: The risk for ACS is increased and direct referral to the ED is warranted.

If the GP or consulting cardiologist disagrees with the recommendation provided by the clinical decision rule, they are allowed to overrule it. The GP is responsible for the patient's follow-up, in line with regular care.

In previous studies, the MHS showed good diagnostic accuracy in ruling out stable coronary artery disease in primary care, but was insufficient to rule out ACS safely.(3, 8, 20, 24) We hypothesise that adding the hs-cTnI-POCT to the MHS will result in a safe and accurate clinical decision rule to rule out ACS in primary care. Even though the aim of the clinical decision rule is to rule out ACS in patients with acute chest pain, it may also reduce missed ACS diagnosis in patients presenting with atypical symptoms.

An ECG is not included in the decision rule, as an ECG device is not available in all general practices in The Netherlands. Nonetheless, GPs with an ECG device available are free to make an ECG and overrule the recommendations of the clinical decision rule as they deem necessary. However, it is important to notice, that an ECG may be normal in 30% of patients

For peer review only - http://bmjopen.bmj.com/site/about/guidelines.xhtml

presenting with non-ST elevated myocardial infarction and is therefore unsuitable for excluding ACS safely.(25)

Safety

Following ESC guidelines the minimum time since onset of chest pain in this study is one hour.(25) Approximately 10% of all patients with acute chest pain in primary care present within one hour since onset of symptoms.(8, 26) Due to the time-depended release of troponin, the decision rule is not applicable for these very early presenters.(25) Hs-cTnI measurements within this time window may give false negative results and therefore wrongfully rule out ACS. Previous studies including early presenters (<3 hours since onset of symptoms), showed decreased sensitivity in patients presenting <1 hour since onset of chest pain.(16, 25-28) In patients presenting 1-3 hours since onset of chest pain, study results are still inconclusive, but using a hs-cTnI - with a cut-off value around the limit of detection, rather than below the 99th percentile - seems safe in combination with clinical evaluation and is recommended in the ESC guideline.(25, 29, 30) To ensure safety and minimise the risk of wrongfully excluding ACS we took the following additional actions. Firstly, patients contacting their GP by telephone with symptoms highly suspicious for ACS are not eligible for inclusion and an ambulance is sent to their home immediately. Patients who are hemodynamically unstable when seen by their GP are excluded as well and require immediate referral. Secondly, the hs-cTnI is embedded in a clinical decision rule, so patients with typical symptoms will be discussed with a cardiologist, even when hs-cTnI measurement is very low. Thirdly, GPs are instructed that the recommendation of the clinical decision rule may be overruled when deemed necessary by the GP or consulting cardiologist. Lastly, a data safety monitoring board is installed to evaluate the safety of the study after 100, 500 and 1000 inclusions.

Primary endpoints

For peer review only - http://bmjopen.bmj.com/site/about/guidelines.xhtml

The study has two primary endpoints. The first primary endpoint is the hospital referral rate for acute chest pain within 24 hours and 6 weeks after inclusion. The second primary endpoint is the diagnostic accuracy of the clinical decision rule. To study the diagnostic accuracy (i.e. sensitivity, negative predictive value), the incidence of ACS and major adverse cardiac events (MACE) at the index consultation, within 6 weeks and 6 months after inclusion will be assessed. MACE is defined as a combined endpoint of ACS, percutaneous coronary intervention, coronary artery bypass grafting, coronary angiography revealing procedurally correctable stenosis managed conservatively and all-cause mortality. (7, 31, 32)

Final diagnosis

The final diagnosis will be made based on GP's medical records, including all letters from hospitals. All cases will be reviewed by an independent endpoint committee consisting of a cardiologist and general practitioner for adjudication of the final diagnosis and the occurrence of MACE. The committee will be blinded for the randomisation and hs-cTnI on presentation. In case of disagreement, a second cardiologist will be consulted. The final diagnosis will be classified as ACS, stable coronary artery disease, cardiac but non coronary disease, non-cardiac chest pain or death. ACS comprises myocardial infarction (ST-segment elevation myocardial infarction (STEMI) and non-ST-segment elevation myocardial infarction (NSTEMI)) and unstable angina pectoris. Myocardial infarction is defined conform the fourth universal definition of myocardial infarction.(33)

A coronary angiogram (CAG) is the golden standard to establish the presence of coronary artery disease. However, CAG is costly and not without risk (e.g. bleeding in approximately 7% and procedure-related myocardial infarction in approximately 6% of patients undergoing CAG).(34, 35) Therefore, we consider it too invasive and essentially unethical to perform a CAG in each patient in this relatively low-risk population. Hence, we will use a delayed reference standard, defined as ACS and MACE at the index consultation, within 6 weeks

For peer review only - http://bmjopen.bmj.com/site/about/guidelines.xhtml

and 6 months after inclusion, to determine the diagnostic accuracy of the decision rule. The delayed reference method has been reported as a valid alternative if a golden standard test is too invasive and the disease is not self-limiting.(36) This method has been used in similar studies before.(7, 20, 32, 37)

Secondary endpoints

Secondary endpoints are the cost-effectiveness of the clinical decision rule, adherence to the recommendations of the clinical decision rule by GPs, the patient's reassurance, the diagnostic accuracy of the GP's gut feeling and the diagnostic accuracy of the HEART-score for all patients with an ECG (Table 2).(31) Finally, subgroup analyses for the primary endpoints will be performed for sex, region, socio-economic status and duration of symptoms.

Table 2. Secondary outcome measures

1.	Cost-effectiveness, consisting of a trial-based cost analysis and a cost-utility analysis (costs per QALY, assessed using the EQ-5D-5L questionnaire).(38, 39)
2.	Adherence to the recommendations of the clinical decision rule by the GP, by comparing the GP's policy with the recommendations of the decision rule.
3.	Reassurance of patients, using the State-Trait Anxiety Inventory after the index consultation.(40)
4.	Diagnostic accuracy of the GP's gut feeling, by comparing the result of the gut feeling questionnaire with the occurrence of MACE within 6 weeks after the index consultation.(41, 42)
5.	Diagnostic accuracy of the HEART-score for all patients with an ECG available for the occurrence of MACE within 6 weeks after the index consultation.(31)
6.	Subgroup analysis for sex, region, socio-economic status (using postal-code) and duration of symptoms.

For peer review only

For peer review only - http://bmjopen.bmj.com/site/about/guidelines.xhtml

The cost-effectiveness of the clinical decision rule will be assessed alongside the trial. The analysis will be performed from a societal perspective. The costs are divided into healthcare costs and non-healthcare costs. Healthcare costs include those of the intervention and other healthcare use during follow-up. The costs of the clinical decision rule will be based on micro-costing including the duration of the GP consultation, medical equipment and materials used. Healthcare use will be assessed by questionnaires at 6 weeks and 6 months follow-up. In this questionnaires, patients will be asked to fill out their total health care utilisation, such as GP visits, outpatient clinic visits, ED visits and hospital admissions for any reason. Reference prices published in the Dutch costing manual will be used to value healthcare use.(43) To calculate non-healthcare costs, patients will be asked to note absence from paid and unpaid work. Productivity costs will be calculated using the friction cost method. QALYs will be calculated from utility scores from the EQ-5D-5L questionnaire administered at baseline, 6 weeks and 6 months follow-up.(38, 39) Finally, costs and QALYs between the control and intervention groups will be compared.

To assess the GP's adherence to the recommendations of the decision rule, the outcome of the decision rule will be compared with the GP's policy as reported by the GP. To assess the effect of the clinical decision rule on patients' reassurance, patients will be asked to fill out the State-Trait Anxiety Inventory after the index consultation.(40) To evaluate the diagnostic accuracy of the GP's gut feeling, GPs will be asked to estimate the likelihood of ACS using the gut feeling questionnaire directly after the index consultation.(41, 42) The outcome of this questionnaire will be compared to the occurrence of MACE within 6 weeks. In all patients with an ECG, the HEART-score will be scored retrospectively based on the information on the inclusion form filled out by the GP. The outcome will be compared to the occurrence of MACE within 6 weeks.(31)

Sample size calculation

For peer review only - http://bmjopen.bmj.com/site/about/guidelines.xhtml

In primary care in the Netherlands, the reported hospital referral rate of patients with chest pain ranges from approximately 40 to 70%.(1, 6) Similarly, a Swiss and Swedish study found referral and diagnostic testing rates of 59% and 43%, respectively.(44, 45) Based on these referral rates, we conservatively assumed a hospital referral rate of 40%. For the primary endpoint, we estimated a 10% decrease in referrals, resulting in 30% referrals, which is in line with a previous study estimating a 10% decrease in referrals when using the MHS.(45, 46) Group sample sizes of 448 (control group) and 896 (intervention group) achieve 80% power to detect the difference of 10% between both groups with a significance level (alpha) of 0.05 using a two-sided Z-test.(47) The sample size is adjusted for a clustering effect with an intra-class correlation coefficient of 0.05 and for 1:2 randomisation.(48) The estimated sample size needed is 500 for the control group and 1000 for the intervention group, accounting for a 10% loss to follow-up.

For peer review only

Statistical analysis

Data will be analysed according to the intention-to-treat principle. Continuous demographic and baseline characteristics will be expressed as mean ± standard deviation and compared using the unpaired Student’s t-test. Categorical demographic and baseline characteristics will be described as frequencies and percentages and compared using the X^2 or Fisher’s exact test. The number of referrals will be presented as percentages and compared using the X^2 or Fisher’s exact test. To assess the diagnostic accuracy of the decision rule, sensitivity, specificity, and predictive values will be calculated. The overall diagnostic accuracy will be assessed by means of the C-statistics. Missingness will be assessed, and if the percentage of missingness is >10%, multiple imputation will be applied. All analyses will be corrected for the correlation within clusters (general practices), using generalised estimating equations. Subgroup analyses will be performed for sex, region, socio-economic status, and duration of symptoms to confirm the applicability of the decision rule for different subgroups.

For peer review only - http://bmjopen.bmj.com/site/about/guidelines.xhtml

Clinical implications

In the Netherlands, GPs are the 'gatekeeper' to the hospital. Therefore, a clinical decision rule for acute chest pain is probably most beneficial in primary care. Based on previous literature, the clinical decision rule presented in this study may decrease hospital referrals of patients with acute chest pain by 10%.(45, 46) Thereby, this strategy may reduce uncertainty for patients and doctors, increase patient satisfaction, decrease (unnecessary) diagnostic tests and relieve pressure on healthcare personnel and the healthcare system. Furthermore, it may lead to a substantial reduction in societal costs as was shown in a previous observational Norwegian study.(17) In the Netherlands, a patient referred to the hospital for acute chest pain on average costs €1580, whereas a primary care consultation plus hs-cTnI-POCT measurement is estimated to cost on average €73.(13, 43) Therefore, if the decision rule would be implemented in 50% of general practices and assuming a 10% reduction in referrals, this could potentially lead to an estimated cost-reduction of €31 million in The Netherlands each year.(49)

Generalisability

The current RCT will compare the clinical decision rule to regular care in general practices in three regions in The Netherlands, including urban and rural areas. After careful instruction on the inclusion criteria, the GPs in these practices will decide on the eligibility of individual patients. Although differences among GPs (e.g. a less experienced GP may include different patients than an experienced GP) may lead to inclusion bias, this situation resembles daily clinical practice. Hence, the results of this study will be generalisable to the rest of The Netherlands and other countries with a similar primary care system, where the GP functions as a gatekeeper (e.g. the UK, Scandinavian countries and Canada). Additionally, we will measure the GP's working experience, which enables us to quantify this potential bias.

Patient involvement

For peer review only - http://bmjopen.bmj.com/site/about/guidelines.xhtml

During the design of the study, the writing of the protocol and the development of patient information materials, a participant of 'Harteraad', a patient advisory council for heart disease, was involved.

Ethics and dissemination

The study will be conducted following the Declaration of Helsinki. The medical ethics committee Leiden-Den Haag-Delft has approved the protocol. Leiden University Medical Center is the sponsor of the study. Written informed consent will be obtained from all participating patients. The trial is registered in the Netherlands Trial Register (NTR) (https://www.trialregister.nl/), NL9525. Accepted on 20-06-2021. Due to the current process of merging the NTR into a new national registry, changes after protocol amendments cannot be made. Therefore we registered the trial a second time on https://www.clinicaltrials.gov/, NCT05827237. A data safety monitoring board (DSMB) has been installed evaluating the safety of the study after 100, 500 and 1000 inclusions.

Results of the study will be shared with professional healthcare workers, participants and the general public. The primary outcomes of the study will be presented in two main papers in scientific, peer reviewed journals. The cost-effectiveness and other secondary outcomes will be shared in separate papers. Findings will be shared at conferences through oral and poster presentations. At the end of the study, all participants will be informed of the study results through email or on paper. End results and interim updates will be shared on the website: www.pobhelp.nl. Participating general practitioners and other stakeholders will be kept up to date through a 3 monthly digital newsletter.

Author's contributions

SB, TB, RW, MN and GD have written the protocol of the study. MB, JM and BR substantially revised the protocol. MA has written the section on cost-effectiveness analyses.

For peer review only

For peer review only - http://bmjopen.bmj.com/site/about/guidelines.xhtml

SB and AP have written this manuscript. All authors read and substantially revised the manuscript.

Funding

This work is supported by ZonMw (project ID 852001907). The Atellica VTLi immunoassay analysers used for the hs-cTnI-POCT measurements in this study are provided by Siemens Healthineers.

Competing interest statement

The authors have no conflicts of interest to declare. Siemens Healthineers provides the immunoassay analysers, cartridges and quality control samples. However, Siemens Healthineers had no role in the design of the study and the results will be analysed and published by the independent researchers.

For peer review only

For peer review only - http://bmjopen.bmj.com/site/about/guidelines.xhtml

References

1. Hoorweg BBN, Willemsen RTA, Cleef L, et al. Frequency of chest pain in primary care, diagnostic tests performed and final diagnoses. Heart. 2017;103:1727-32.

2. Bosner S, Becker A, Haasenritter J, et al. Chest pain in primary care: epidemiology and pre-work-up probabilities. Eur J Gen Pract. 2009;15:141-6.

3. Bosner S, Haasenritter J, Becker A, et al. Ruling out coronary artery disease in primary care: development and validation of a simple prediction rule. CMAJ. 2010;182:1295-300.

4. Terkelsen CJ, Sørensen JT, Maeng M, et al. System delay and mortality among patients with STEMI treated with primary percutaneous coronary intervention. JAMA. 2010;304(7):763-71.

5. Harskamp R, van Peet P, Bont J, et al. The conundrum of acute chest pain in general practice: a nationwide survey in The Netherlands. BJGP Open. 2018;2:1-10.

6. Bruins Slot MH, Rutten FH, van der Heijden GJ, et al. Diagnosing acute coronary syndrome in primary care: comparison of the physicians' risk estimation and a clinical decision rule. Fam Pract. 2011;28(3):323-8.

7. Poldervaart JM, Reitsma JB, Backus BE, et al. Effect of Using the HEART Score in Patients With Chest Pain in the Emergency Department. Ann Intern Med. 2017;166(10):689.

8. Schols AMR, Willemsen RTA, Bonten TN, et al. A Nationwide Flash-Mob Study for Suspected Acute Coronary Syndrome. Ann Fam Med. 2019;17(4):296-303.

9. Chiang CH, Chiang CH, Pickering JW, et al. Performance of the European Society of Cardiology 0/1-Hour, 0/2-Hour, and 0/3-Hour Algorithms for Rapid Triage of Acute Myocardial Infarction : An International Collaborative Meta-analysis. Ann Intern Med. 2022;175(1):101-13.

10. Pines JM, Pollack CV, Jr., Diercks DB, et al. The association between emergency department crowding and adverse cardiovascular outcomes in patients with chest pain. Acad Emerg Med. 2009;16(7):617-25.

For peer review only - http://bmjopen.bmj.com/site/about/guidelines.xhtml

11. Sun BC, Hsia RY, Weiss RE, et al. Effect of emergency department crowding on outcomes of admitted patients. Ann Emerg Med. 2013;61(6):605-11 e6.

12. Rasouli HR, Esfahani AA, Nobakht M, et al. Outcomes of Crowding in Emergency Departments; a Systematic Review. Arch Acad Emerg Med. 2019;7(1):e52.

13. van Dongen DN, Ottervanger JP, Tolsma R, et al. In-Hospital Healthcare Utilization, Outcomes, and Costs in Pre-Hospital-Adjudicated Low-Risk Chest-Pain Patients. Appl Health Econ Health Policy. 2019;17(6):875-82.

14. Pickering JW, Than MP, Cullen L, et al. Rapid Rule-out of Acute Myocardial Infarction With a Single High-Sensitivity Cardiac Troponin T Measurement Below the Limit of Detection: A Collaborative Meta-analysis. Ann Intern Med. 2017;166(10):715-24.

15. Kip MMA, Koffijberg H, Moesker MJ, et al. The cost-utility of point-of-care troponin testing to diagnose acute coronary syndrome in primary care. BMC Cardiovasc Disord. 2017;17(1):213.

16. Johannessen TR, Vallersnes OM, Halvorsen S, et al. Pre-hospital One-Hour Troponin in a Low-Prevalence Population of Acute Coronary Syndrome: OUT-ACS study. Open Heart. 2020;7(2).

17. Johannessen TR, Halvorsen S, Atar D, et al. Cost-effectiveness of a rule-out algorithm of acute myocardial infarction in low-risk patients: emergency primary care versus hospital setting. BMC Health Serv Res. 2022;22(1):1274.

18. Castor EDC. Castor Electronic Data Capture 2019 [online]. 2019. Available from: https://castoredc.com.

19. Auwerda A, Bouma M, Bruins Slot M, et al. NHG standaard Acuut coronair syndroom [online]. 2022. Available from: https://richtlijnen.nhg.org/standaarden/acuut-coronair-syndroom.

20. Haasenritter J, Bosner S, Vaucher P, et al. Ruling out coronary heart disease in primary care: external validation of a clinical prediction rule. Br J Gen Pract. 2012;62:415-21.

For peer review only

For peer review only - http://bmjopen.bmj.com/site/about/guidelines.xhtml

21. Apple FS, Schulz K, Schmidt CW, et al. Determination of sex-specific 99th percentile upper reference limits for a point of care high sensitivity cardiac troponin I assay. Clin Chem Lab Med. 2021;59(9):1574-8.

22. Christenson RH, Frenk LDS, de Graaf HJ, et al. Point-of-Care: Roadmap for Analytical Characterization and Validation of a High-Sensitivity Cardiac Troponin I Assay in Plasma and Whole Blood Matrices. J Appl Lab Med. 2022;7(4):971-88.

23. Apple FS, Smith SW, Greenslade JH, et al. Single High-Sensitivity Point of Care Whole Blood Cardiac Troponin I Measurement to Rule Out Acute Myocardial Infarction at Low Risk. Circulation. 2022;146(25):1918-1929.

24. Harskamp R, Laeven SC, Himmelreich J, et al. Chest pain in general practice: a systematic review of prediction rules. BMJ open. 2018;9:1-10.

25. Collet JP, Thiele H, Barbato E, et al. 2020 ESC Guidelines for the management of acute coronary syndromes in patients presenting without persistent ST-segment elevation. Eur Heart J. 2020;41(37):3495-3497.

26. Andersen CF, Bang C, Lauridsen KG, et al. Single troponin measurement to rule-out acute myocardial infarction in early presenters. Int J Cardiol. 2021;341:15-21.

27. Ljung L, Reichard C, Hagerman P, et al. Sensitivity of undetectable level of high-sensitivity troponin T at presentation in a large non-ST-segment elevation myocardial infarction cohort of early presenters. Int J Cardiol. 2019;284:6-11.

28. Body R, Carley S, McDowell G, et al. Rapid exclusion of acute myocardial infarction in patients with undetectable troponin using a high-sensitivity assay. J Am Coll Cardiol. 2011;58(13):1332-9.

29. Crea F, Jaffe AS, Collinson PO, et al. Should the 1h algorithm for rule in and rule out of acute myocardial infarction be used universally? Eur Heart J. 2016;37(44):3316-23.

30. Apple FS, Collinson PO, Kavsak PA, et al. Getting Cardiac Troponin Right: Appraisal of the 2020 European Society of Cardiology Guidelines for the Management of Acute Coronary Syndromes in Patients Presenting without Persistent ST-Segment Elevation by the

For peer review only

For peer review only - http://bmjopen.bmj.com/site/about/guidelines.xhtml

International Federation of Clinical Chemistry and Laboratory Medicine Committee on Clinical Applications of Cardiac Bio-Markers. Clin Chem. 2021;67(5):730-5.

31. Backus BE, Six AJ, Kelder JC, et al. A prospective validation of the HEART score for chest pain patients at the emergency department. Int J Cardiol.. 2013;168(3):2153-8.

32. van Dongen DN, Fokkert MJ, Tolsma RT, et al. Accuracy of pre-hospital HEART score risk classification using point of care versus high sensitive troponin in suspected NSTE-ACS. Am J Emerg Med. 2020;38(8):1616-20.

33. Thygesen K, Alpert JS, Jaffe AS, et al. Fourth universal definition of myocardial infarction (2018). Eur Heart J. 2019;40(3):237-69.

34. Mehta SR, Cannon CP, Fox KA, et al. Routine vs selective invasive strategies in patients with acute coronary syndromes: a collaborative meta-analysis of randomized trials. JAMA. 2005;293(23):2908-17.

35. Fanning JP, Nyong J, Scott IA, et al. Routine invasive strategies versus selective invasive strategies for unstable angina and non-ST elevation myocardial infarction in the stent era. Cochrane Database Syst Rev. 2016;2016(5):Cd004815.

36. Knottnerus JA, Muris JW. Assessment of the accuracy of diagnostic tests: the cross-sectional study. J Clin Epidemiol. 2003;56(11):1118-28.

37. Tolsma RT, Fokkert MJ, van Dongen DN, et al. Referral decisions based on a pre-hospital HEART score in suspected non-ST-elevation acute coronary syndrome: final results of the FamouS Triage study. Eur Heart J Acute Cardiovasc Care. 2021;11(2):160-169.

38. Herdman M, Gudex C, Lloyd A, et al. Development and preliminary testing of the new five-level version of EQ-5D (EQ-5D-5L). Qual Life Res. 2011;20(10):1727-36.

39. Versteegh MM, Vermeulen KM, Evers SMAA, et al. Dutch Tariff for the Five-Level Version of EQ-5D. Value Health. 2016;19(4):343-52.

40. Spielberger, C. D. (1983). Manual for the State-Trait Anxiety Inventory STAI. Palo Alto, CA: Mind Garden

For peer review only

For peer review only - http://bmjopen.bmj.com/site/about/guidelines.xhtml

41. Stolper CF, Van de Wiel MWJ, De Vet HCW, et al. Family physicians' diagnostic gut feelings are measurable: construct validation of a questionnaire. BMC Fam Pract. 2013;14(1).

42. Barais M, Fossard E, Dany A, et al. Accuracy of the general practitioner's sense of alarm when confronted with dyspnoea and/or chest pain: a prospective observational study. BMJ Open. 2020;10(2):e034348.

43. Kanters TA, Bouwmans CAM, van der Linden N, et al. Update of the Dutch manual for costing studies in health care. PLoS One. 2017;12(11):e0187477.

44. Verdon F, Herzig L, Burnand B, et al. Chest pain in daily practice: occurrence, causes and management. Swiss med wkly. 2008;138:340-7.

45. Nilsson S, Andersson A, Janzon M, et al. Cost consequences of point-of-care troponin T testing in a Swedish primary health care setting. Scand J Prim Health Care. 2014;32(4):241-7.

46. Haasenritter J, Donner-Banzhoff N, Bösner S. Chest pain for coronary heart disease in general practice: clinical judgement and a clinical decision rule. Br J Gen Pract. 2015;65(640):e748-53.

47. Donner A, Klar N. Design and Analysis of Cluster Randomization Trials in Health Research. London: Arnold Publishers; 2000.

48. Smeeth L, Ng ES-W. Intraclass correlation coefficients for cluster randomized trials in primary care: data from the MRC Trial of the Assessment and management of Older People in the Community. Control Clin Trials. 2002;23:409-21.

49. Grol R, Dalhuijsen J, Thomas S, et al. Attributes of clinical guidelines that influence use of guidelines in general practice: observational study. MJ. 1998;317(7162):858-61.

For peer review only

For peer review only - http://bmjopen.bmj.com/site/about/guidelines.xhtml

468 Figure 1. Flowchart of the clinical decision rule

469

For peer review only

For peer review only - http://bmjopen.bmj.com/site/about/guidelines.xhtml

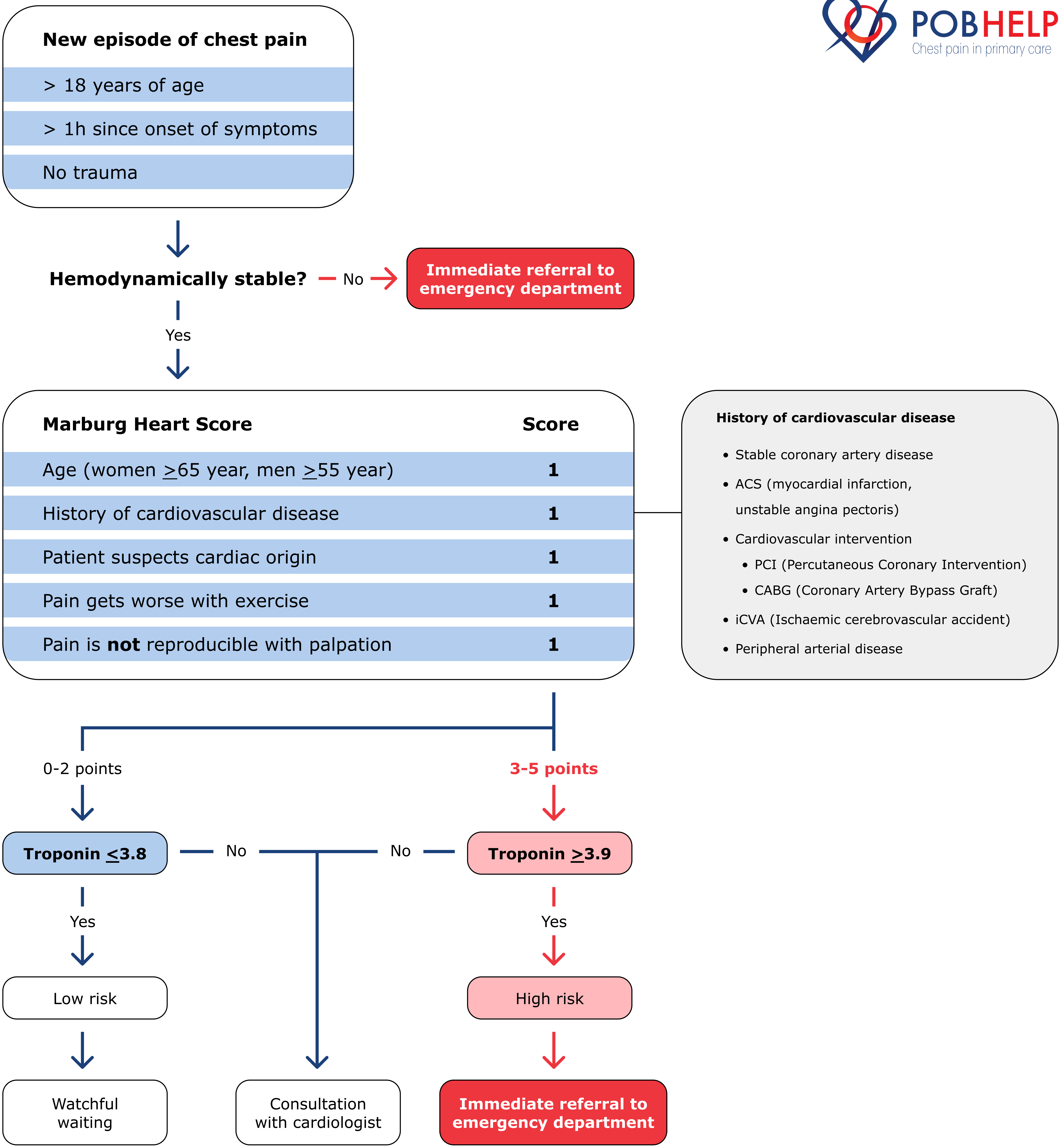
New episode of chest pain
> 18 years of age
> 1h since onset of symptoms
No trauma
Hemodynamically stable?
No
Immediate referral to emergency department
Yes
Marburg Heart Score
Score
Age (women >65 year, men >55 year)
1
History of cardiovascular disease
1
Patient suspects cardiac origin
1
Pain gets worse with exercise
1
Pain is **not** reproducible with palpation
1
History of cardiovascular disease
• Stable coronary artery disease
• ACS (myocardial infarction, unstable angina pectoris)
• Cardiovascular intervention
• PCI (Percutaneous Coronary Intervention)
• CABG (Coronary Artery Bypass Graft)
• iCVA (Ischaemic cerebrovascular accident)
• Peripheral arterial disease
0-2 points
3-5 points
Troponin <3.8
No
No
Troponin >3.9
Yes
Yes
Low risk
High risk
Watchful waiting
Consultation with cardiologist
Immediate referral to emergency department

SPIRIT

STANDARD PROTOCOL ITEMS: RECOMMENDATIONS FOR INTERVENTIONAL TRIALS

SPIRIT 2013 Checklist: Recommended items to address in a clinical trial protocol and related documents*

Section/item	ItemNo	Description	Page number
**Administrative information**			
Title	1	Descriptive title identifying the study design, population, interventions, and, if applicable, trial acronym	1
Trial registration	2a	Trial identifier and registry name. If not yet registered, name of intended registry	1
	2b	All items from the World Health Organization Trial Registration Data Set	Throughout protocol
Protocol version	3	Date and version identifier	All pages
Funding	4	Sources and types of financial, material, and other support	3
Roles and responsibilities	5a	Names, affiliations, and roles of protocol contributors	2,3
	5b	Name and contact information for the trial sponsor	3
	5c	Role of study sponsor and funders, if any, in study design; collection, management, analysis, and interpretation of data; writing of the report; and the decision to submit the report for publication, including whether they will have ultimate authority over any of these activities	23
	5d	Composition, roles, and responsibilities of the coordinating centre, steering committee, endpoint adjudication committee, data management team, and other individuals or groups overseeing the trial, if applicable (see Item 21a for data monitoring committee)	Throughout protocol
**Introduction**			
Background and rationale	6a	Description of research question and justification for undertaking the trial, including summary of relevant studies (published and unpublished) examining benefits and harms for each intervention	10-12
	6b	Explanation for choice of comparators	x

For peer review only

Objectives	7	Specific objectives or hypotheses	12, 13
Trial design	8	Description of trial design including type of trial (eg, parallel group, crossover, factorial, single group), allocation ratio, and framework (eg, superiority, equivalence, noninferiority, exploratory)	13
**Methods: Participants, interventions, and outcomes**			
Study setting	9	Description of study settings (eg, community clinic, academic hospital) and list of countries where data will be collected. Reference to where list of study sites can be obtained	13, 14
Eligibility criteria	10	Inclusion and exclusion criteria for participants. If applicable, eligibility criteria for study centres and individuals who will perform the interventions (eg, surgeons, psychotherapists)	13, 14
Interventions	11a	Interventions for each group with sufficient detail to allow replication, including how and when they will be administered	16-18
	11b	Criteria for discontinuing or modifying allocated interventions for a given trial participant (eg, drug dose change in response to harms, participant request, or improving/worsening disease)	22
	11c	Strategies to improve adherence to intervention protocols, and any procedures for monitoring adherence (eg, drug tablet return, laboratory tests)	n/a
	11d	Relevant concomitant care and interventions that are permitted or prohibited during the trial	n/a
Outcomes	12	Primary, secondary, and other outcomes, including the specific measurement variable (eg, systolic blood pressure), analysis metric (eg, change from baseline, final value, time to event), method of aggregation (eg, median, proportion), and time point for each outcome. Explanation of the clinical relevance of chosen efficacy and harm outcomes is strongly recommended	9-11<br>18-21
Participant timeline	13	Time schedule of enrolment, interventions (including any run-ins and washouts), assessments, and visits for participants. A schematic diagram is highly recommended (see Figure)	21, 22
Sample size	14	Estimated number of participants needed to achieve study objectives and how it was determined, including clinical and statistical assumptions supporting any sample size calculations	14, 15

For peer review only - http://bmjopen.bmj.com/site/about/guidelines.xhtml

Recruitment	15	Strategies for achieving adequate participant enrolment to reach target sample size	30
**Methods: Assignment of interventions (for controlled trials)**			
Allocation:			
Sequence generation	16a	Method of generating the allocation sequence (eg, computer-generated random numbers), and list of any factors for stratification. To reduce predictability of a random sequence, details of any planned restriction (eg, blocking) should be provided in a separate document that is unavailable to those who enrol participants or assign interventions	21
Allocation concealment mechanism	16b	Mechanism of implementing the allocation sequence (eg, central telephone; sequentially numbered, opaque, sealed envelopes), describing any steps to conceal the sequence until interventions are assigned	n/a
Implementation	16c	Who will generate the allocation sequence, who will enrol participants, and who will assign participants to interventions	21
Blinding (masking)	17a	Who will be blinded after assignment to interventions (eg, trial participants, care providers, outcome assessors, data analysts), and how	n/a
	17b	If blinded, circumstances under which unblinding is permissible, and procedure for revealing a participant's allocated intervention during the trial	n/a
**Methods: Data collection, management, and analysis**			
Data collection methods	18a	Plans for assessment and collection of outcome, baseline, and other trial data, including any related processes to promote data quality (eg, duplicate measurements, training of assessors) and a description of study instruments (eg, questionnaires, laboratory tests) along with their reliability and validity, if known. Reference to where data collection forms can be found, if not in the protocol	9-11
	18b	Plans to promote participant retention and complete follow-up, including list of any outcome data to be collected for participants who discontinue or deviate from intervention protocols	28-29

For peer review only - http://bmjopen.bmj.com/site/about/guidelines.xhtml

Data management	19	Plans for data entry, coding, security, and storage, including any related processes to promote data quality (eg, double data entry; range checks for data values). Reference to where details of data management procedures can be found, if not in the protocol	31, 32
Statistical methods	20a	Statistical methods for analysing primary and secondary outcomes. Reference to where other details of the statistical analysis plan can be found, if not in the protocol	26, 27
	20b	Methods for any additional analyses (eg, subgroup and adjusted analyses)	26, 27
	20c	Definition of analysis population relating to protocol non-adherence (eg, as randomised analysis), and any statistical methods to handle missing data (eg, multiple imputation)	27
**Methods: Monitoring**			
Data monitoring	21a	Composition of data monitoring committee (DMC); summary of its role and reporting structure; statement of whether it is independent from the sponsor and competing interests; and reference to where further details about its charter can be found, if not in the protocol. Alternatively, an explanation of why a DMC is not needed	25
	21b	Description of any interim analyses and stopping guidelines, including who will have access to these interim results and make the final decision to terminate the trial	n/a
Harms	22	Plans for collecting, assessing, reporting, and managing solicited and spontaneously reported adverse events and other unintended effects of trial interventions or trial conduct	24, 25
Auditing	23	Frequency and procedures for auditing trial conduct, if any, and whether the process will be independent from investigators and the sponsor	32
**Ethics and dissemination**			
Research ethics approval	24	Plans for seeking research ethics committee/institutional review board (REC/IRB) approval	14

Protocol amendments	25	Plans for communicating important protocol modifications (eg, changes to eligibility criteria, outcomes, analyses) to relevant parties (eg, investigators, REC/IRBs, trial participants, trial registries, journals, regulators)	32
Consent or assent	26a	Who will obtain informed consent or assent from potential trial participants or authorised surrogates, and how (see Item 32)	27-30
	26b	Additional consent provisions for collection and use of participant data and biological specimens in ancillary studies, if applicable	n/a
Confidentiality	27	How personal information about potential and enrolled participants will be collected, shared, and maintained in order to protect confidentiality before, during, and after the trial	31, 32
Declaration of interests	28	Financial and other competing interests for principal investigators for the overall trial and each study site	n/a
Access to data	29	Statement of who will have access to the final trial dataset, and disclosure of contractual agreements that limit such access for investigators	23
Ancillary and post-trial care	30	Provisions, if any, for ancillary and post-trial care, and for compensation to those who suffer harm from trial participation	31
Dissemination policy	31a	Plans for investigators and sponsor to communicate trial results to participants, healthcare professionals, the public, and other relevant groups (eg, via publication, reporting in results databases, or other data sharing arrangements), including any publication restrictions	33
	31b	Authorship eligibility guidelines and any intended use of professional writers	x
	31c	Plans, if any, for granting public access to the full protocol, participant-level dataset, and statistical code	Trial registration
**Appendices**			
Informed consent materials	32	Model consent form and other related documentation given to participants and authorised surrogates	appendix
Biological specimens	33	Plans for collection, laboratory evaluation, and storage of biological specimens for genetic or molecular analysis in the current trial and for future use in ancillary studies, if applicable	n/a

For peer review only - http://bmjopen.bmj.com/site/about/guidelines.xhtml

*It is strongly recommended that this checklist be read in conjunction with the SPIRIT 2013 Explanation & Elaboration for important clarification on the items. Amendments to the protocol should be tracked and dated. The SPIRIT checklist is copyrighted by the SPIRIT Group under the Creative Commons "Attribution-NonCommercial-NoDerivs 3.0 Unported" license.

For peer review only

BMJ Open

Ruling out acute coronary syndrome in primary care with a clinical decision rule and a capillary, high-sensitive troponin I point of care test: Study protocol of a diagnostic RCT in the Netherlands (POB HELP)

Journal:	*BMJ Open*
Manuscript ID	bmjopen-2023-071822.R2
Article Type:	Protocol
Date Submitted by the Author:	12-May-2023
Complete List of Authors:	van den Bulk, Simone; Leiden Universitair Medisch Centrum, Public Health and primary Care<br>Petrus, Annelieke; Leiden Universitair Medisch Centrum, Public Health and primary Care<br>Willemsen, Robert ; Maastricht University, Department of Family Medicine<br>Boogers, Mark; Leiden Universitair Medisch Centrum, Cardiology<br>Meeder, Joan; VieCuri Medisch Centrum voor Noord-Limburg, Cardiology<br>Rahel, Braim; VieCuri Medisch Centrum voor Noord-Limburg, Cardiology<br>van den Akker-van Marle, Elske; Leiden University Medical Center, Biomedical Data Sciences<br>Numans, Mattijs; Leiden Universitair Medisch Centrum, Public Health and primary Care<br>Dinant, Geert-Jan; Maastricht University, Department of Family Medicine<br>Bonten, Tobias N.; Leiden Universitair Medisch Centrum, Public Health and primary Care
**Primary Subject Heading**:	General practice / Family practice
Secondary Subject Heading:	Cardiovascular medicine, Diagnostics
Keywords:	Myocardial infarction < CARDIOLOGY, PRIMARY CARE, Decision Making, ACCIDENT & EMERGENCY MEDICINE

SCHOLARONE™
Manuscripts

Ruling out acute coronary syndrome in primary care with a clinical decision rule and a capillary, high-sensitive troponin I point of care test: Study protocol of a diagnostic RCT in the Netherlands (POB HELP)

S. van den Bulk MD[1], A.H.J. Petrus MD PhD[1], R.T. Willemsen MD PhD[2], M.J. Boogers[3], J.G. Meeder[4], B.M. Rahel[4], M.E. van den Akker-van Marle[5], PhD, M.E. Numans MD PhD[1], G.J. Dinant MD PhD[2], T.N. Bonten MD PhD[1]

For peer review only

Word count: 3604

Addresses of authors

1 Department of Public Health and Primary Care
Leiden University Medical Center
Post zone V0-P
Postbus 9600
2300 RC Leiden, The Netherlands

2 Faculty of Health, Medicine and Life Sciences
Maastricht University Medical Center+

PO BOX 616
6200 MD Maastricht, The Netherlands

3 Department of Cardiology
Leiden University Medical Center
Post zone V0-P
Postbus 9600
2300 RC Leiden, The Netherlands

4 Department of Cardiology
VieCuri Medical center for Noord-Limburg
PO BOX 1926
5900 BX Venlo, The Netherlands

5 Department of Biomedical Data Sciences,
unit Medical Decision Making
Leiden University Medical Center
Post zone J10-S
Postbus 9600
2300 RC Leiden, The Netherlands

Corresponding author
Simone van den Bulk, MD
@ s.van_den_bulk@lumc.nl
T: +31 625043440

Department of Public Health and Primary Care
Leiden University Medical Center
Post zone V0-P
Postbus 9600
2300 RC Leiden, The Netherland

For peer review only - http://bmjopen.bmj.com/site/about/guidelines.xhtml

Abstract

Introduction: Chest pain is a common reason for consultation in primary care. To rule out acute coronary syndrome (ACS), general practitioners (GP) refer 40-70% of patients with chest pain to the emergency department (ED). Only 10-20% of those referred, are diagnosed with ACS. A clinical decision rule, including a high-sensitive cardiac troponin-I point-of-care test (hs-cTnI-POCT), may safely rule out ACS in primary care. Being able to safely rule out ACS at the GP level reduces referrals and thereby alleviates the burden on the ED. Moreover, prompt feedback to the patients may reduce anxiety and stress.

Methods and analysis: The POB HELP study is a clustered randomised controlled diagnostic trial investigating the (cost-)effectiveness and diagnostic accuracy of a primary care decision rule for acute chest pain, consisting of the Marburg Heart Score combined with a hs-cTnI-POCT (limit of detection 1.6 ng/L, 99th percentile 23 ng/L, cut-off value between negative and positive used in this study 3.8 ng/L). General practices are 2:1 randomised to the intervention group (clinical decision rule) or control group (regular care). In total 1500 patients with acute chest pain are planned to be included by GPs in three regions in The Netherlands. Primary endpoints are the number of hospital referrals and the diagnostic accuracy of the decision rule 24 hours, 6 weeks and 6 months after inclusion.

Ethics and dissemination: The medical ethics committee Leiden-Den Haag-Delft (the Netherlands) has approved this trial. Written informed consent will be obtained from all participating patients. The results of this trial will be disseminated in one main paper and additional papers on secondary endpoints and subgroup analyses.

Trial registration number: Netherlands Trial Register (NL9525) and www.clinicaltrials.gov (NCT05827237).

For peer review only

Strengths and limitations of this study (5)

- The study design is a randomised controlled diagnostic trial, which prospectively evaluates a clinical decision rule, including a high-sensitive cardiac troponin I (hs-cTnI) point-of-care test, to rule out acute coronary syndrome (ACS) in primary care.
- The study includes a cost-effectiveness analysis, to evaluate the efficiency of the clinical decision rule and the impact on the healthcare system.
- The Atellica VTLi hs-cTnI analyser provides reliable hs-cTnI measurements within only 8 minutes in capillary blood obtained by a fingerstick.
- Inclusion is based on clinical suspicion of ACS by general practitioners, which reflects daily clinical practice, but may lead to inclusion bias.
- Patients with an onset of chest pain <1 hour are excluded, because hs-cTnI measurement within this time window may be false negative due to time-dependent troponin release.

For peer review only

For peer review only - http://bmjopen.bmj.com/site/about/guidelines.xhtml

Introduction

Chest pain is a common reason for consultation in primary care.(1, 2) In 4-7% of patients presenting with chest pain, the pain is caused by acute coronary syndrome (ACS).(1-3) Early identification and treatment of patients with ACS are important to avoid cardiac morbidity and mortality.(4) However, it is challenging for general practitioners (GPs) to distinguish ACS from other – less acute and life-threatening – causes of chest pain, as they have to rely on symptoms and clinical signs only.(5) Consequently, GPs refer approximately 40-70% of patients presenting with chest pain to the hospital for further examination.(1, 6)

Following referral, only 10-20% of the patients are diagnosed with ACS.(7-9) The relatively high number of referrals puts a high burden on patients and may lead to potentially avoidable adverse patient outcomes due to the risks associated with (unnecessary) diagnostic tests and overcrowding of emergency departments (ED).(10-12) In addition, a high referral rate puts a lot of pressure on the healthcare system. Finally, evaluation of patients in the ED is costly, which has a significant impact on society as well.(13) Therefore, measures to improve the referral efficiency of patients with acute chest pain is crucial.

A clinical decision rule may help GPs to safely rule out ACS and reduce hospital referrals. Previous studies in ED settings showed that a single high-sensitive cardiac troponin-I (hs-cTnI) measurement with or without a risk score is safe and effective to rule out ACS.(7, 9, 14) However, due to the low incidence of ACS in primary care, results from ED settings are not generalisable to primary care settings. Although use of a troponin point of care test (POCT) in primary care reduced the number of referrals in both a model-based and an observational study, the impact has yet to be studied in clinical practice.(15-17)

Therefore, this clustered randomised controlled diagnostic trial (RCT) aims to evaluate the impact of a primary care decision rule for acute chest pain, consisting of a risk stratification score combined with a hs-cTnI-POCT on the number of hospital referrals, and to assess its diagnostic accuracy. In addition, secondary endpoints including cost-effectiveness will be assessed.

For peer review only - http://bmjopen.bmj.com/site/about/guidelines.xhtml

Methods and analysis

Study design

The POB HELP study is a clustered diagnostic RCT. The study will be conducted in general practices in 3 regions in the Netherlands (Leiden, Maastricht, Venlo). General practices will be randomised to the intervention or control group in a 2:1 ratio. Randomisation is stratified for the 3 regions to ensure even distribution of rural and urban areas. Randomisation is done by Castor Electronic Data Capture using a variable block randomisation method.(18) Patient inclusion started on 18th August 2021 and the study is planned to end on 1st July 2024.

Study population

Patients visiting their GP, or visited by their GP, for acute chest pain symptoms in which the GP suspects an ACS, are eligible for inclusion. Patients who contact their GP by telephone with acute chest pain highly suspicious for ACS are not eligible for inclusion. For these patients an ambulance is sent directly to the home address by the doctor's assistant, in accordance with usual care. Exclusion criteria are: age <18 years, trauma preceding the pain, inability to speak Dutch or understand the informed consent form, onset of chest pain within less than one hour and hemodynamic instability (Table 1). Patients who match the eligibility criteria and provide informed consent are included in the study.

Table 1. In- and exclusion criteria

Inclusion	Exclusion
≥18 years of age	< 1 hour since onset of symptoms
Acute chest pain	Inability to speak Dutch or understand Dutch
Seen by the general practitioner	Hemodynamic instability

Regular care

For peer review only

For peer review only - http://bmjopen.bmj.com/site/about/guidelines.xhtml

In general practices randomised to the control group, included patients will receive standard care in accordance with the Dutch GP guideline for ACS.(19) Standard care consists of a medical history and physical examination by the GP. In addition, an electrocardiogram (ECG) can be performed, however an ECG device is not available in many general practices in the Netherlands. Based on this information, the GP will decide to either reassure the patient ("watchful waiting"), consult a cardiologist, or refer the patient to the hospital. The GP is responsible for the follow-up of the patient.

Intervention

For patients included in general practices randomised to the intervention group, the GP will apply a clinical decision rule consisting of the Marburg Heart Score (MHS) and a hs-cTnI-POCT (Figure 1).(3, 20, 21)

The MHS consists of a 5-item score and each positive item results in 1 point: 1) Age and sex (male ≥ 55 years, female ≥ 65 years), 2) History of cardiovascular disease (i.e. stable coronary artery disease, prior myocardial infarction, coronary intervention, ischaemic cerebrovascular accident or peripheral atherosclerosis), 3) Patient assumes a cardiac origin of the chest pain, 4) Pain worsening with exercise and 5) Pain not reproducible with palpation. The ACS risk is considered low for patients with a MHS of ≤ 2 points and high for patients with ≥ 3 points.(3, 20)

The hs-cTnI measurement is performed using the Siemens Atellica VTLi immunoassay analyser.(21) The analyser is easy to use and guides the user step by step through the process. Prior to the start of the study, the staff, including the doctor's assistants and at least one GP per general practice, is trained by a study employee. A study employee is available during office hours to provide assistance by telephone when necessary. Blood is obtained by a fingerstick, which makes it highly suitable for a pre-hospital setting. The droplet of blood is applied to a cartridge using a capillary transfer device. The turn-around time from the

For peer review only

For peer review only - http://bmjopen.bmj.com/site/about/guidelines.xhtml

application of the droplet of blood to the test result is approximately 8 minutes. The limit of detection is 1.6 ng/L and the limit of quantitation is 8.9 ng/L (at 10% CV) and 3.7 ng/L (at 20% CV).(22) The 99th percentile overall, for men, and for women are respectively 23, 27 and 18 ng/L. A hs-cTnI level ≤ 3.8 ng/L is considered very low and is used as a cut-off in this study to exclude ACS.(23)

The combination of the MHS and the hs-cTnI-POCT measurement results in 3 possible recommendations (Figure 1):

1) MHS ≤ 2 points and hs-cTnI-POCT ≤ 3.8 ng/L: ACS is safely excluded and watchful waiting is recommended.

2) either MHS ≤ 2 points and hs-cTnI-POCT ≥ 3.9 ng/L, or MHS ≥ 3 and hs-cTnI-POCT ≤ 3.8 ng/L: ACS cannot be excluded, consultation with a cardiologist is recommended.

3) MHS ≥ 3 and hs-cTnI-POCT ≥ 3.9 ng/L: The risk for ACS is increased and direct referral to the ED is warranted.

If the GP or consulting cardiologist disagrees with the recommendation provided by the clinical decision rule, they are allowed to overrule it. The GP is responsible for the patient's follow-up, in line with regular care.

In previous studies, the MHS showed good diagnostic accuracy in ruling out stable coronary artery disease in primary care, but was insufficient to rule out ACS safely.(3, 8, 20, 24) We hypothesise that adding the hs-cTnI-POCT to the MHS will result in a safe and accurate clinical decision rule to rule out ACS in primary care. Even though the aim of the clinical decision rule is to rule out ACS in patients with acute chest pain, it may also reduce missed ACS diagnosis in patients presenting with atypical symptoms.

An ECG is not included in the decision rule, as an ECG device is not available in all general practices in The Netherlands. Nonetheless, GPs with an ECG device available are free to make an ECG and overrule the recommendations of the clinical decision rule as they deem necessary. However, it is important to notice, that an ECG may be normal in 30% of patients

For peer review only - http://bmjopen.bmj.com/site/about/guidelines.xhtml

For peer review only

presenting with non-ST elevated myocardial infarction and is therefore unsuitable for excluding ACS safely.(25)

Safety

Following ESC guidelines the minimum time since onset of chest pain in this study is one hour.(25) Approximately 10% of all patients with acute chest pain in primary care present within one hour since onset of symptoms.(8, 26) Due to the time-depended release of troponin, the decision rule is not applicable for these very early presenters.(25) Hs-cTnI measurements within this time window may give false negative results and therefore wrongfully rule out ACS. Previous studies including early presenters (<3 hours since onset of symptoms), showed decreased sensitivity in patients presenting <1 hour since onset of chest pain.(16, 25-28) In patients presenting 1-3 hours since onset of chest pain, study results are still inconclusive, but using a hs-cTnI - with a cut-off value around the limit of detection, rather than below the 99th percentile - seems safe in combination with clinical evaluation and is recommended in the ESC guideline.(25, 29, 30) To ensure safety and minimise the risk of wrongfully excluding ACS we took the following additional actions. Firstly, patients contacting their GP by telephone with symptoms highly suspicious for ACS are not eligible for inclusion and an ambulance is sent to their home immediately. Patients who are hemodynamically unstable when seen by their GP are excluded as well and require immediate referral. Secondly, the hs-cTnI is embedded in a clinical decision rule, so patients with typical symptoms will be discussed with a cardiologist, even when hs-cTnI measurement is very low. Thirdly, GPs are instructed that the recommendation of the clinical decision rule may be overruled when deemed necessary by the GP or consulting cardiologist. Lastly, a data safety monitoring board is installed to evaluate the safety of the study after 100, 500 and 1000 inclusions.

Primary endpoints

The study has two primary endpoints. The first primary endpoint is the hospital referral rate for acute chest pain within 24 hours and 6 weeks after inclusion. The second primary endpoint is

For peer review only - http://bmjopen.bmj.com/site/about/guidelines.xhtml

the diagnostic accuracy of the clinical decision rule. To study the diagnostic accuracy (i.e. sensitivity, negative predictive value), the incidence of ACS and major adverse cardiac events (MACE) at the index consultation, within 6 weeks and 6 months after inclusion will be assessed. MACE is defined as a combined endpoint of ACS, percutaneous coronary intervention, coronary artery bypass grafting, coronary angiography revealing procedurally correctable stenosis managed conservatively and all-cause mortality. (7, 31, 32)

Final diagnosis

The final diagnosis will be made based on GP's medical records, including all letters from hospitals. All cases will be reviewed by an independent endpoint committee consisting of a cardiologist and general practitioner for adjudication of the final diagnosis and the occurrence of MACE. The committee will be blinded for the randomisation and hs-cTnI on presentation. In case of disagreement, a second cardiologist will be consulted. The final diagnosis will be classified as ACS, stable coronary artery disease, cardiac but non coronary disease, non-cardiac chest pain or death. ACS comprises myocardial infarction (ST-segment elevation myocardial infarction (STEMI) and non-ST-segment elevation myocardial infarction (NSTEMI)) and unstable angina pectoris. Myocardial infarction is defined conform the fourth universal definition of myocardial infarction.(33)

For peer review only

A coronary angiogram (CAG) is the golden standard to establish the presence of coronary artery disease. However, CAG is costly and not without risk (e.g. bleeding in approximately 7% and procedure-related myocardial infarction in approximately 6% of patients undergoing CAG).(34, 35) Therefore, we consider it too invasive and essentially unethical to perform a CAG in each patient in this relatively low-risk population. Hence, we will use a delayed reference standard, defined as ACS and MACE at the index consultation, within 6 weeks and 6 months after inclusion, to determine the diagnostic accuracy of the decision rule. The delayed reference method has been reported as a valid alternative if a golden standard test is

For peer review only - http://bmjopen.bmj.com/site/about/guidelines.xhtml

too invasive and the disease is not self-limiting.(36) This method has been used in similar studies before.(7, 20, 32, 37)

Secondary endpoints

Secondary endpoints are the cost-effectiveness of the clinical decision rule, adherence to the recommendations of the clinical decision rule by GPs, the patient's reassurance, the diagnostic accuracy of the GP's gut feeling and the diagnostic accuracy of the HEART-score for all patients with an ECG (Table 2).(31) Finally, subgroup analyses for the primary endpoints will be performed for sex, region, socio-economic status and duration of symptoms.

For peer review only

Table 2. Secondary outcome measures

1.	Cost-effectiveness, consisting of a trial-based cost analysis and a cost-utility analysis (costs per QALY, assessed using the EQ-5D-5L questionnaire).(38, 39)
2.	Adherence to the recommendations of the clinical decision rule by the GP, by comparing the GP's policy with the recommendations of the decision rule.
3.	Reassurance of patients, using the State-Trait Anxiety Inventory after the index consultation.(40)
4.	Diagnostic accuracy of the GP's gut feeling, by comparing the result of the gut feeling questionnaire with the occurrence of MACE within 6 weeks after the index consultation.(41, 42)
5.	Diagnostic accuracy of the HEART-score for all patients with an ECG available for the occurrence of MACE within 6 weeks after the index consultation.(31)
6.	Subgroup analysis for the primary outcomes for sex, region, socio-economic status (using postal-code) and duration of symptoms.

The cost-effectiveness of the clinical decision rule will be assessed alongside the trial. The analysis will be performed from a societal perspective. The costs are divided into healthcare

For peer review only - http://bmjopen.bmj.com/site/about/guidelines.xhtml

costs and non-healthcare costs. Healthcare costs include those of the intervention and other healthcare use during follow-up. The costs of the clinical decision rule will be based on micro-costing including the duration of the GP consultation, medical equipment and materials used. Healthcare use will be assessed by questionnaires at 6 weeks and 6 months follow-up. In this questionnaires, patients will be asked to fill out their total health care utilisation, such as GP visits, outpatient clinic visits, ED visits and hospital admissions for any reason. Reference prices published in the Dutch costing manual will be used to value healthcare use.(43) To calculate non-healthcare costs, patients will be asked to note absence from paid and unpaid work. Productivity costs will be calculated using the friction cost method. QALYs will be calculated from utility scores from the EQ-5D-5L questionnaire administered at baseline, 6 weeks and 6 months follow-up.(38, 39) Finally, costs and QALYs between the control and intervention groups will be compared.

To assess the GP's adherence to the recommendations of the decision rule, the outcome of the decision rule will be compared with the GP's policy as reported by the GP. To assess the effect of the clinical decision rule on patients' reassurance, patients will be asked to fill out the State-Trait Anxiety Inventory after the index consultation.(40) To evaluate the diagnostic accuracy of the GP's gut feeling, GPs will be asked to estimate the likelihood of ACS using the gut feeling questionnaire directly after the index consultation.(41, 42) The outcome of this questionnaire will be compared to the occurrence of MACE within 6 weeks. In all patients with an ECG, the HEART-score will be scored retrospectively based on the information on the inclusion form filled out by the GP. The outcome will be compared to the occurrence of MACE within 6 weeks.(31)

Sample size calculation

In primary care in the Netherlands, the reported hospital referral rate of patients with chest pain ranges from approximately 40 to 70%.(1, 6) Similarly, a Swiss and Swedish study found referral and diagnostic testing rates of 59% and 43%, respectively.(44, 45) Based on these

For peer review only - http://bmjopen.bmj.com/site/about/guidelines.xhtml

referral rates, we conservatively assumed a hospital referral rate of 40%. For the primary endpoint, we estimated a 10% decrease in referrals, resulting in 30% referrals, which is in line with a previous study estimating a 10% decrease in referrals when using the MHS.(45, 46) Group sample sizes of 448 (control group) and 896 (intervention group) achieve 80% power to detect the difference of 10% between both groups with a significance level (alpha) of 0.05 using a two-sided Z-test.(47) The sample size is adjusted for a clustering effect with an intra-class correlation coefficient of 0.05 and for 1:2 randomisation.(48) The estimated sample size needed is 500 for the control group and 1000 for the intervention group, accounting for a 10% loss to follow-up.

Statistical analysis

Data will be analysed according to the intention-to-treat principle. Continuous demographic and baseline characteristics will be expressed as mean ± standard deviation and compared using the unpaired Student's t-test. Categorical demographic and baseline characteristics will be described as frequencies and percentages and compared using the X^2 or Fisher's exact test. The number of referrals will be presented as percentages and compared using the X^2 or Fisher's exact test. To assess the diagnostic accuracy of the decision rule, sensitivity, specificity, and predictive values will be calculated. The overall diagnostic accuracy will be assessed by means of the C-statistics. Missingness will be assessed, and if the percentage of missingness is >10%, multiple imputation will be applied. All analyses will be corrected for the correlation within clusters (general practices), using generalised estimating equations. Subgroup analyses will be performed for sex, region, socio-economic status, and duration of symptoms to confirm the applicability of the decision rule for different subgroups.

Clinical implications

In the Netherlands, GPs are the 'gatekeeper' to the hospital. Therefore, a clinical decision rule for acute chest pain is probably most beneficial in primary care. Based on previous literature, the clinical decision rule presented in this study may decrease hospital referrals of patients

For peer review only - http://bmjopen.bmj.com/site/about/guidelines.xhtml

with acute chest pain by 10%.(45, 46) Thereby, this strategy may reduce uncertainty for patients and doctors, increase patient satisfaction, decrease (unnecessary) diagnostic tests and relieve pressure on healthcare personnel and the healthcare system. Furthermore, it may lead to a substantial reduction in societal costs as was shown in a previous observational Norwegian study.(17) In the Netherlands, a patient referred to the hospital for acute chest pain on average costs €1580, whereas a primary care consultation plus hs-cTnI-POCT measurement is estimated to cost on average €73.(13, 43) Therefore, if the decision rule would be implemented in 50% of general practices and assuming a 10% reduction in referrals, this could potentially lead to an estimated cost-reduction of €31 million in The Netherlands each year.(49)

Generalisability

The current RCT will compare the clinical decision rule to regular care in general practices in three regions in The Netherlands, including urban and rural areas. After careful instruction on the inclusion criteria, the GPs in these practices will decide on the eligibility of individual patients. Although differences among GPs (e.g. a less experienced GP may include different patients than an experienced GP) may lead to inclusion bias, this situation resembles daily clinical practice. Hence, the results of this study will be generalisable to the rest of The Netherlands and other countries with a similar primary care system, where the GP functions as a gatekeeper (e.g. the UK, Scandinavian countries and Canada). Additionally, we will measure the GP's working experience, which enables us to quantify this potential bias.

Patient involvement

During the design of the study, the writing of the protocol and the development of patient information materials, a participant of 'Harteraad', a patient advisory council for heart disease, was involved.

Ethics and dissemination

For peer review only

The study will be conducted following the Declaration of Helsinki. The medical ethics committee Leiden-Den Haag-Delft has approved the protocol. Leiden University Medical Center is the sponsor of the study. Written informed consent will be obtained from all participating patients. The trial is registered in the Netherlands Trial Register (NTR) (https://www.trialregister.nl/), NL9525. Accepted on 20-06-2021. Due to the current process of merging the NTR into a new national registry, changes after protocol amendments cannot be made. Therefore we registered the trial a second time on https://www.clinicaltrials.gov/, NCT05827237. A data safety monitoring board (DSMB) has been installed evaluating the safety of the study after 100, 500 and 1000 inclusions.

Results of the study will be shared with professional healthcare workers, participants and the general public. The primary outcomes of the study will be presented in two main papers in scientific, peer reviewed journals. The cost-effectiveness and other secondary outcomes will be shared in separate papers. Findings will be shared at conferences through oral and poster presentations. At the end of the study, all participants will be informed of the study results through email or on paper. End results and interim updates will be shared on the website: www.pobhelp.nl. Participating general practitioners and other stakeholders will be kept up to date through a 3 monthly digital newsletter.

Author's contributions

SB, TB, RW, MN and GD have written the protocol of the study. MB, JM and BR substantially revised the protocol. MA has written the section on cost-effectiveness analyses. SB and AP have written this manuscript. All authors read and substantially revised the manuscript. All authors gave final approval of the published version.

Funding

For peer review only

325 This work is supported by ZonMw (project ID 852001907). The Atellica VTLi immunoassay analysers used for the hs-cTnI-POCT measurements in this study are provided by Siemens Healthineers.

Competing interest statement

330 The authors have no conflicts of interest to declare. Siemens Healthineers provides the immunoassay analysers, cartridges and quality control samples. However, Siemens Healthineers had no role in the design of the study and the results will be analysed and published by the independent researchers.

For peer review only

For peer review only - http://bmjopen.bmj.com/site/about/guidelines.xhtml

References

1. Hoorweg BBN, Willemsen RTA, Cleef L, et al. Frequency of chest pain in primary care, diagnostic tests performed and final diagnoses. Heart. 2017;103:1727-32.

2. Bosner S, Becker A, Haasenritter J, et al. Chest pain in primary care: epidemiology and pre-work-up probabilities. Eur J Gen Pract. 2009;15:141-6.

3. Bosner S, Haasenritter J, Becker A, et al. Ruling out coronary artery disease in primary care: development and validation of a simple prediction rule. CMAJ. 2010;182:1295-300.

4. Terkelsen CJ, Sørensen JT, Maeng M, et al. System delay and mortality among patients with STEMI treated with primary percutaneous coronary intervention. JAMA. 2010;304(7):763-71.

5. Harskamp R, van Peet P, Bont J, et al. The conundrum of acute chest pain in general practice: a nationwide survey in The Netherlands. BJGP Open. 2018;2:1-10.

6. Bruins Slot MH, Rutten FH, van der Heijden GJ, et al. Diagnosing acute coronary syndrome in primary care: comparison of the physicians' risk estimation and a clinical decision rule. Fam Pract. 2011;28(3):323-8.

7. Poldervaart JM, Reitsma JB, Backus BE, et al. Effect of Using the HEART Score in Patients With Chest Pain in the Emergency Department. Ann Intern Med. 2017;166(10):689.

8. Schols AMR, Willemsen RTA, Bonten TN, et al. A Nationwide Flash-Mob Study for Suspected Acute Coronary Syndrome. Ann Fam Med. 2019;17(4):296-303.

9. Chiang CH, Chiang CH, Pickering JW, et al. Performance of the European Society of Cardiology 0/1-Hour, 0/2-Hour, and 0/3-Hour Algorithms for Rapid Triage of Acute Myocardial Infarction : An International Collaborative Meta-analysis. Ann Intern Med. 2022;175(1):101-13.

10. Pines JM, Pollack CV, Jr., Diercks DB, et al. The association between emergency department crowding and adverse cardiovascular outcomes in patients with chest pain. Acad Emerg Med. 2009;16(7):617-25.

For peer review only

11. Sun BC, Hsia RY, Weiss RE, et al. Effect of emergency department crowding on outcomes of admitted patients. Ann Emerg Med. 2013;61(6):605-11 e6.

12. Rasouli HR, Esfahani AA, Nobakht M, et al. Outcomes of Crowding in Emergency Departments; a Systematic Review. Arch Acad Emerg Med. 2019;7(1):e52.

13. van Dongen DN, Ottervanger JP, Tolsma R, et al. In-Hospital Healthcare Utilization, Outcomes, and Costs in Pre-Hospital-Adjudicated Low-Risk Chest-Pain Patients. Appl Health Econ Health Policy. 2019;17(6):875-82.

14. Pickering JW, Than MP, Cullen L, et al. Rapid Rule-out of Acute Myocardial Infarction With a Single High-Sensitivity Cardiac Troponin T Measurement Below the Limit of Detection: A Collaborative Meta-analysis. Ann Intern Med. 2017;166(10):715-24.

15. Kip MMA, Koffijberg H, Moesker MJ, et al. The cost-utility of point-of-care troponin testing to diagnose acute coronary syndrome in primary care. BMC Cardiovasc Disord. 2017;17(1):213.

16. Johannessen TR, Vallersnes OM, Halvorsen S, et al. Pre-hospital One-Hour Troponin in a Low-Prevalence Population of Acute Coronary Syndrome: OUT-ACS study. Open Heart. 2020;7(2).

17. Johannessen TR, Halvorsen S, Atar D, et al. Cost-effectiveness of a rule-out algorithm of acute myocardial infarction in low-risk patients: emergency primary care versus hospital setting. BMC Health Serv Res. 2022;22(1):1274.

18. Castor EDC. Castor Electronic Data Capture 2019 [online]. 2019. Available from: https://castoredc.com.

19. Auwerda A, Bouma M, Bruins Slot M, et al. NHG standaard Acuut coronair syndroom [online]. 2022. Available from: https://richtlijnen.nhg.org/standaarden/acuut-coronair-syndroom.

20. Haasenritter J, Bosner S, Vaucher P, et al. Ruling out coronary heart disease in primary care: external validation of a clinical prediction rule. Br J Gen Pract. 2012;62:415-21.

For peer review only

For peer review only - http://bmjopen.bmj.com/site/about/guidelines.xhtml

21. Apple FS, Schulz K, Schmidt CW, et al. Determination of sex-specific 99th percentile upper reference limits for a point of care high sensitivity cardiac troponin I assay. Clin Chem Lab Med. 2021;59(9):1574-8.

22. Christenson RH, Frenk LDS, de Graaf HJ, et al. Point-of-Care: Roadmap for Analytical Characterization and Validation of a High-Sensitivity Cardiac Troponin I Assay in Plasma and Whole Blood Matrices. J Appl Lab Med. 2022;7(4):971-88.

23. Apple FS, Smith SW, Greenslade JH, et al. Single High-Sensitivity Point of Care Whole Blood Cardiac Troponin I Measurement to Rule Out Acute Myocardial Infarction at Low Risk. Circulation. 2022;146(25):1918-1929.

24. Harskamp R, Laeven SC, Himmelreich J, et al. Chest pain in general practice: a systematic review of prediction rules. BMJ open. 2018;9:1-10.

25. Collet JP, Thiele H, Barbato E, et al. 2020 ESC Guidelines for the management of acute coronary syndromes in patients presenting without persistent ST-segment elevation. Eur Heart J. 2020;41(37):3495-3497.

26. Andersen CF, Bang C, Lauridsen KG, et al. Single troponin measurement to rule-out acute myocardial infarction in early presenters. Int J Cardiol. 2021;341:15-21.

27. Ljung L, Reichard C, Hagerman P, et al. Sensitivity of undetectable level of high-sensitivity troponin T at presentation in a large non-ST-segment elevation myocardial infarction cohort of early presenters. Int J Cardiol. 2019;284:6-11.

28. Body R, Carley S, McDowell G, et al. Rapid exclusion of acute myocardial infarction in patients with undetectable troponin using a high-sensitivity assay. J Am Coll Cardiol. 2011;58(13):1332-9.

29. Crea F, Jaffe AS, Collinson PO, et al. Should the 1h algorithm for rule in and rule out of acute myocardial infarction be used universally? Eur Heart J. 2016;37(44):3316-23.

30. Apple FS, Collinson PO, Kavsak PA, et al. Getting Cardiac Troponin Right: Appraisal of the 2020 European Society of Cardiology Guidelines for the Management of Acute Coronary Syndromes in Patients Presenting without Persistent ST-Segment Elevation by the

For peer review only

For peer review only - http://bmjopen.bmj.com/site/about/guidelines.xhtml

International Federation of Clinical Chemistry and Laboratory Medicine Committee on Clinical Applications of Cardiac Bio-Markers. Clin Chem. 2021;67(5):730-5.

31. Backus BE, Six AJ, Kelder JC, et al. A prospective validation of the HEART score for chest pain patients at the emergency department. Int J Cardiol.. 2013;168(3):2153-8.

32. van Dongen DN, Fokkert MJ, Tolsma RT, et al. Accuracy of pre-hospital HEART score risk classification using point of care versus high sensitive troponin in suspected NSTE-ACS. Am J Emerg Med. 2020;38(8):1616-20.

33. Thygesen K, Alpert JS, Jaffe AS, et al. Fourth universal definition of myocardial infarction (2018). Eur Heart J. 2019;40(3):237-69.

34. Mehta SR, Cannon CP, Fox KA, et al. Routine vs selective invasive strategies in patients with acute coronary syndromes: a collaborative meta-analysis of randomized trials. JAMA. 2005;293(23):2908-17.

35. Fanning JP, Nyong J, Scott IA, et al. Routine invasive strategies versus selective invasive strategies for unstable angina and non-ST elevation myocardial infarction in the stent era. Cochrane Database Syst Rev. 2016;2016(5):Cd004815.

36. Knottnerus JA, Muris JW. Assessment of the accuracy of diagnostic tests: the cross-sectional study. J Clin Epidemiol. 2003;56(11):1118-28.

37. Tolsma RT, Fokkert MJ, van Dongen DN, et al. Referral decisions based on a pre-hospital HEART score in suspected non-ST-elevation acute coronary syndrome: final results of the FamouS Triage study. Eur Heart J Acute Cardiovasc Care. 2021;11(2):160-169.

38. Herdman M, Gudex C, Lloyd A, et al. Development and preliminary testing of the new five-level version of EQ-5D (EQ-5D-5L). Qual Life Res. 2011;20(10):1727-36.

39. Versteegh MM, Vermeulen KM, Evers SMAA, et al. Dutch Tariff for the Five-Level Version of EQ-5D. Value Health. 2016;19(4):343-52.

40. Spielberger, C. D. (1983). Manual for the State-Trait Anxiety Inventory STAI. Palo Alto, CA: Mind Garden

For peer review only

For peer review only - http://bmjopen.bmj.com/site/about/guidelines.xhtml

41. Stolper CF, Van de Wiel MWJ, De Vet HCW, et al. Family physicians' diagnostic gut feelings are measurable: construct validation of a questionnaire. BMC Fam Pract. 2013;14(1).

42. Barais M, Fossard E, Dany A, et al. Accuracy of the general practitioner's sense of alarm when confronted with dyspnoea and/or chest pain: a prospective observational study. BMJ Open. 2020;10(2):e034348.

43. Kanters TA, Bouwmans CAM, van der Linden N, et al. Update of the Dutch manual for costing studies in health care. PLoS One. 2017;12(11):e0187477.

44. Verdon F, Herzig L, Burnand B, et al. Chest pain in daily practice: occurrence, causes and management. Swiss med wkly. 2008;138:340-7.

45. Nilsson S, Andersson A, Janzon M, et al. Cost consequences of point-of-care troponin T testing in a Swedish primary health care setting. Scand J Prim Health Care. 2014;32(4):241-7.

46. Haasenritter J, Donner-Banzhoff N, Bösner S. Chest pain for coronary heart disease in general practice: clinical judgement and a clinical decision rule. Br J Gen Pract. 2015;65(640):e748-53.

47. Donner A, Klar N. Design and Analysis of Cluster Randomization Trials in Health Research. London: Arnold Publishers; 2000.

48. Smeeth L, Ng ES-W. Intraclass correlation coefficients for cluster randomized trials in primary care: data from the MRC Trial of the Assessment and management of Older People in the Community. Control Clin Trials. 2002;23:409-21.

49. Grol R, Dalhuijsen J, Thomas S, et al. Attributes of clinical guidelines that influence use of guidelines in general practice: observational study. MJ. 1998;317(7162):858-61.

For peer review only

For peer review only - http://bmjopen.bmj.com/site/about/guidelines.xhtml

464 Figure 1. Flowchart of the clinical decision rule

465

For peer review only

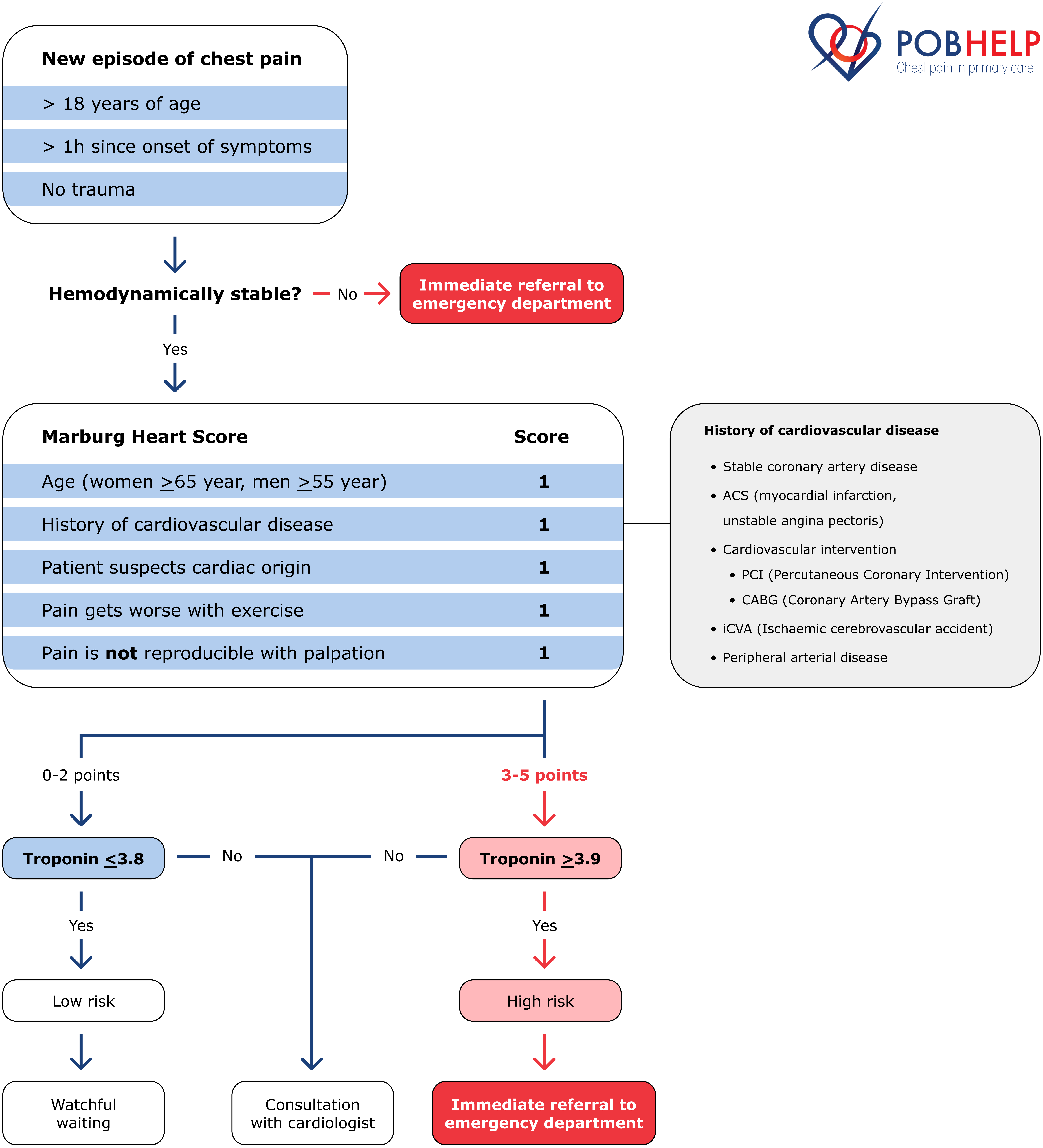
POBHELP
Chest pain in primary care
New episode of chest pain
> 18 years of age
> 1h since onset of symptoms
No trauma
Hemodynamically stable?
No
Immediate referral to emergency department
Yes
Marburg Heart Score
Score
Age (women >65 year, men >55 year)
1
History of cardiovascular disease
1
Patient suspects cardiac origin
1
Pain gets worse with exercise
1
Pain is not reproducible with palpation
1
History of cardiovascular disease
Stable coronary artery disease
ACS (myocardial infarction, unstable angina pectoris)
Cardiovascular intervention
PCI (Percutaneous Coronary Intervention)
CABG (Coronary Artery Bypass Graft)
iCVA (Ischaemic cerebrovascular accident)
Peripheral arterial disease
0-2 points
3-5 points
Troponin <3.8
No
No
Troponin >3.9
Yes
Yes
Low risk
High risk
Watchful waiting
Consultation with cardiologist
Immediate referral to emergency department

SPIRIT

STANDARD PROTOCOL ITEMS: RECOMMENDATIONS FOR INTERVENTIONAL TRIALS

SPIRIT 2013 Checklist: Recommended items to address in a clinical trial protocol and related documents*

Section/item	ItemNo	Description	Page number
**Administrative information**			
Title	1	Descriptive title identifying the study design, population, interventions, and, if applicable, trial acronym	1
Trial registration	2a	Trial identifier and registry name. If not yet registered, name of intended registry	1
	2b	All items from the World Health Organization Trial Registration Data Set	Throughout protocol
Protocol version	3	Date and version identifier	All pages
Funding	4	Sources and types of financial, material, and other support	3
Roles and responsibilities	5a	Names, affiliations, and roles of protocol contributors	2,3
	5b	Name and contact information for the trial sponsor	3
	5c	Role of study sponsor and funders, if any, in study design; collection, management, analysis, and interpretation of data; writing of the report; and the decision to submit the report for publication, including whether they will have ultimate authority over any of these activities	23
	5d	Composition, roles, and responsibilities of the coordinating centre, steering committee, endpoint adjudication committee, data management team, and other individuals or groups overseeing the trial, if applicable (see Item 21a for data monitoring committee)	Throughout protocol
**Introduction**			
Background and rationale	6a	Description of research question and justification for undertaking the trial, including summary of relevant studies (published and unpublished) examining benefits and harms for each intervention	10-12
	6b	Explanation for choice of comparators	x

For peer review only

Objectives	7	Specific objectives or hypotheses	12, 13
Trial design	8	Description of trial design including type of trial (eg, parallel group, crossover, factorial, single group), allocation ratio, and framework (eg, superiority, equivalence, noninferiority, exploratory)	13
**Methods: Participants, interventions, and outcomes**			
Study setting	9	Description of study settings (eg, community clinic, academic hospital) and list of countries where data will be collected. Reference to where list of study sites can be obtained	13, 14
Eligibility criteria	10	Inclusion and exclusion criteria for participants. If applicable, eligibility criteria for study centres and individuals who will perform the interventions (eg, surgeons, psychotherapists)	13, 14
Interventions	11a	Interventions for each group with sufficient detail to allow replication, including how and when they will be administered	16-18
	11b	Criteria for discontinuing or modifying allocated interventions for a given trial participant (eg, drug dose change in response to harms, participant request, or improving/worsening disease)	22
	11c	Strategies to improve adherence to intervention protocols, and any procedures for monitoring adherence (eg, drug tablet return, laboratory tests)	n/a
	11d	Relevant concomitant care and interventions that are permitted or prohibited during the trial	n/a
Outcomes	12	Primary, secondary, and other outcomes, including the specific measurement variable (eg, systolic blood pressure), analysis metric (eg, change from baseline, final value, time to event), method of aggregation (eg, median, proportion), and time point for each outcome. Explanation of the clinical relevance of chosen efficacy and harm outcomes is strongly recommended	9-11<br>18-21
Participant timeline	13	Time schedule of enrolment, interventions (including any run-ins and washouts), assessments, and visits for participants. A schematic diagram is highly recommended (see Figure)	21, 22
Sample size	14	Estimated number of participants needed to achieve study objectives and how it was determined, including clinical and statistical assumptions supporting any sample size calculations	14, 15

For peer review only - http://bmjopen.bmj.com/site/about/guidelines.xhtml

Recruitment	15	Strategies for achieving adequate participant enrolment to reach target sample size	30
**Methods: Assignment of interventions (for controlled trials)**			
Allocation:			
Sequence generation	16a	Method of generating the allocation sequence (eg, computer-generated random numbers), and list of any factors for stratification. To reduce predictability of a random sequence, details of any planned restriction (eg, blocking) should be provided in a separate document that is unavailable to those who enrol participants or assign interventions	21
Allocation concealment mechanism	16b	Mechanism of implementing the allocation sequence (eg, central telephone; sequentially numbered, opaque, sealed envelopes), describing any steps to conceal the sequence until interventions are assigned	n/a
Implementation	16c	Who will generate the allocation sequence, who will enrol participants, and who will assign participants to interventions	21
Blinding (masking)	17a	Who will be blinded after assignment to interventions (eg, trial participants, care providers, outcome assessors, data analysts), and how	n/a
	17b	If blinded, circumstances under which unblinding is permissible, and procedure for revealing a participant's allocated intervention during the trial	n/a
**Methods: Data collection, management, and analysis**			
Data collection methods	18a	Plans for assessment and collection of outcome, baseline, and other trial data, including any related processes to promote data quality (eg, duplicate measurements, training of assessors) and a description of study instruments (eg, questionnaires, laboratory tests) along with their reliability and validity, if known. Reference to where data collection forms can be found, if not in the protocol	9-11
	18b	Plans to promote participant retention and complete follow-up, including list of any outcome data to be collected for participants who discontinue or deviate from intervention protocols	28-29

For peer review only - http://bmjopen.bmj.com/site/about/guidelines.xhtml

Data management	19	Plans for data entry, coding, security, and storage, including any related processes to promote data quality (eg, double data entry; range checks for data values). Reference to where details of data management procedures can be found, if not in the protocol	31, 32
Statistical methods	20a	Statistical methods for analysing primary and secondary outcomes. Reference to where other details of the statistical analysis plan can be found, if not in the protocol	26, 27
	20b	Methods for any additional analyses (eg, subgroup and adjusted analyses)	26, 27
	20c	Definition of analysis population relating to protocol non-adherence (eg, as randomised analysis), and any statistical methods to handle missing data (eg, multiple imputation)	27
**Methods: Monitoring**			
Data monitoring	21a	Composition of data monitoring committee (DMC); summary of its role and reporting structure; statement of whether it is independent from the sponsor and competing interests; and reference to where further details about its charter can be found, if not in the protocol. Alternatively, an explanation of why a DMC is not needed	25
	21b	Description of any interim analyses and stopping guidelines, including who will have access to these interim results and make the final decision to terminate the trial	n/a
Harms	22	Plans for collecting, assessing, reporting, and managing solicited and spontaneously reported adverse events and other unintended effects of trial interventions or trial conduct	24, 25
Auditing	23	Frequency and procedures for auditing trial conduct, if any, and whether the process will be independent from investigators and the sponsor	32
**Ethics and dissemination**			
Research ethics approval	24	Plans for seeking research ethics committee/institutional review board (REC/IRB) approval	14

For peer review only - http://bmjopen.bmj.com/site/about/guidelines.xhtml

Protocol amendments	25	Plans for communicating important protocol modifications (eg, changes to eligibility criteria, outcomes, analyses) to relevant parties (eg, investigators, REC/IRBs, trial participants, trial registries, journals, regulators)	32
Consent or assent	26a	Who will obtain informed consent or assent from potential trial participants or authorised surrogates, and how (see Item 32)	27-30
	26b	Additional consent provisions for collection and use of participant data and biological specimens in ancillary studies, if applicable	n/a
Confidentiality	27	How personal information about potential and enrolled participants will be collected, shared, and maintained in order to protect confidentiality before, during, and after the trial	31, 32
Declaration of interests	28	Financial and other competing interests for principal investigators for the overall trial and each study site	n/a
Access to data	29	Statement of who will have access to the final trial dataset, and disclosure of contractual agreements that limit such access for investigators	23
Ancillary and post-trial care	30	Provisions, if any, for ancillary and post-trial care, and for compensation to those who suffer harm from trial participation	31
Dissemination policy	31a	Plans for investigators and sponsor to communicate trial results to participants, healthcare professionals, the public, and other relevant groups (eg, via publication, reporting in results databases, or other data sharing arrangements), including any publication restrictions	33
	31b	Authorship eligibility guidelines and any intended use of professional writers	x
	31c	Plans, if any, for granting public access to the full protocol, participant-level dataset, and statistical code	Trial registration
**Appendices**			
Informed consent materials	32	Model consent form and other related documentation given to participants and authorised surrogates	appendix
Biological specimens	33	Plans for collection, laboratory evaluation, and storage of biological specimens for genetic or molecular analysis in the current trial and for future use in ancillary studies, if applicable	n/a

For peer review only - http://bmjopen.bmj.com/site/about/guidelines.xhtml

*It is strongly recommended that this checklist be read in conjunction with the SPIRIT 2013 Explanation & Elaboration for important clarification on the items. Amendments to the protocol should be tracked and dated. The SPIRIT checklist is copyrighted by the SPIRIT Group under the Creative Commons "Attribution-NonCommercial-NoDerivs 3.0 Unported" license.

For peer review only
